# Supplementary material for: Two-dimensional organic-inorganic hybrid perovskite quantum-well nanowires enabled by directional noncovalent intermolecular interactions
Source: Nat Commun. 2025 Mar 27;16:2997. doi: 10.1038/s41467-025-58166-x (PMC11950231; doi:10.1038/s41467-025-58166-x)
Supplement: Supplementary file 1 — Supplementary Information [file 41467_2025_58166_MOESM1_ESM.pdf]

Supplementary information for

2D Organic-Inorganic Hybrid Perovskite Quantum-Well Nanowires Enabled by Directional Noncovalent Intermolecular Interactions

*Meng Zhang[1 #], Leyang Jin[1 #], Tianhao Zhang[1], Xiaofan Jiang[1], Mingyuan Li[1], Yan Guan[1], and Yongping Fu[1]**

[1] Beijing National Laboratory for Molecular Science, College of Chemistry and Molecular Engineering, Peking University, Beijing 100871, China

[#] These authors contribute equally

* Corresponding author: Email: yfu@pku.edu.cn

Supplementary Movie 1, Real-time video of the growth of $(PMA)_2PbI_4$ nanowires, PMA^+ = phenylmethylammonium. The size of the window is 380 μm × 280 μm.

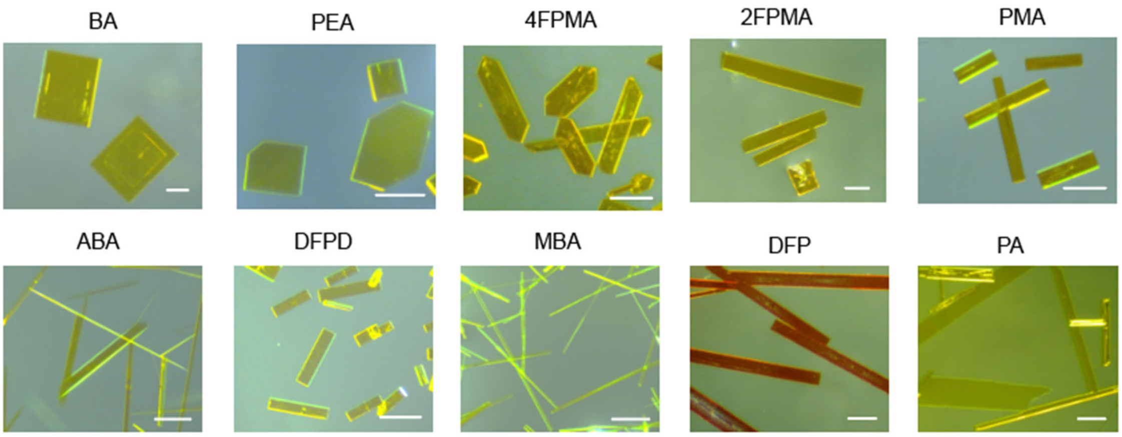

Supplementary Fig. 1 Optical images of 2D perovskite microcrystals with various LA^+ cations, showing different morphologies. All scale bars are 100 μm.

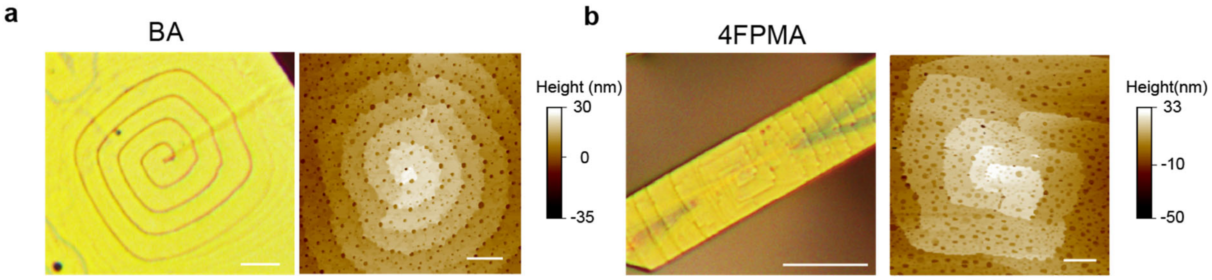

Supplementary Fig. 2 Additional examples showing spiral cores. a, b, Optical and atomic force microscope (AFM) images of $(BA)PbI_4$ (**a**) and $(4FPMA)_2PbI_4$ (**b**) microcrystals showing the screw dislocation cores. Scale bars in the optical images are 20 μm. Scale bars in the AFM images are 1 μm and 2 μm for $(BA)PbI_4$ and $(4FPMA)_2PbI_4$, respectively.

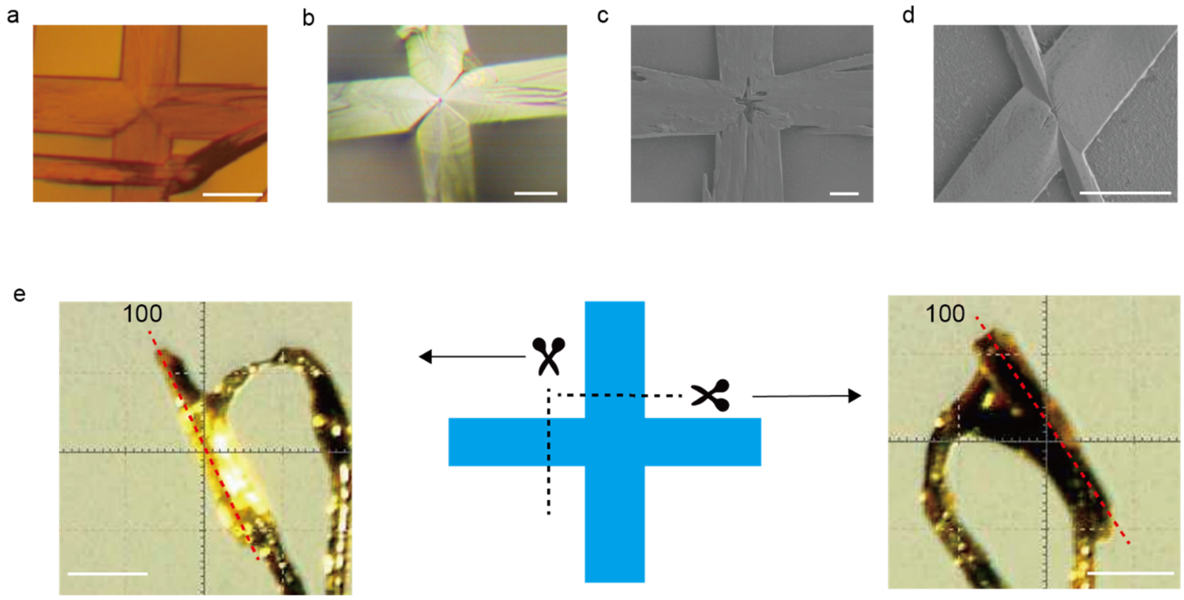

Supplementary Fig. 3 Branching crystal growth in certain 2D perovskites. a, b, Optical images of $(MBA)_2PbI_4$ (**a**) and $(PMA)_2PbI_4$ (**b**) crystals with 4 branches. The Scale bars are 25 μm. **c, d,** SEM images of $(MBA)_2PbI_4$ crystals with 4 branches. The Scale bars are 10 μm. **e,** Optical images of the cleaved $(MBA)_2PbI_4$ branches mounted on the diffractor, confirming that each branch follows the same crystallographic direction, corresponding to stronger intermolecular interactions. The Scale bars are 100 μm.

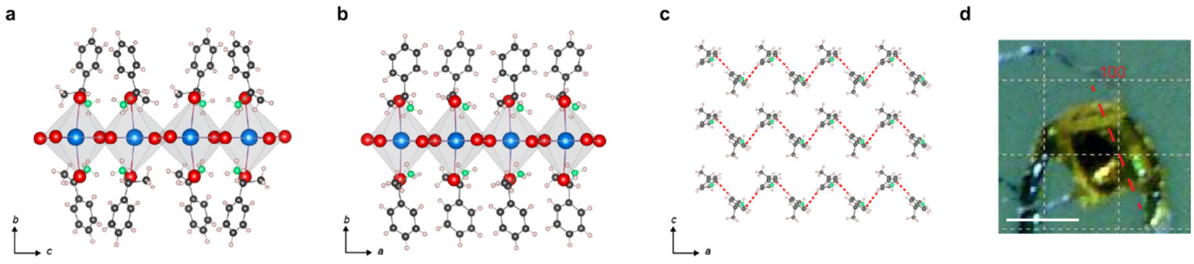

Supplementary Fig. 4 Crystal structures and single-crystal X-ray diffraction characterization of $(MBA)_2PbI_4$. a, b, Crystal structures of $(MBA)_2PbI_4$ viewed along the *a*-

axis (**a**) and along the *c*-axis (**b**). $(MBA)_2PbI_4$ adopts a space group $P2_12_12_1$, with a = 8.89 Å, b = 28.87 Å, c = 9.31 Å, and α = β = γ = 90°. **c**, The arrangement of the MBA^+ cations viewed along the *b*-axis (out-of-plane direction), showing directional intermolecular C-H···π interactions along the *a*-axis. **d,** Optical image of a $(MBA)_2PbI_4$ crystal mounted on the diffractor, confirming that the longer axis corresponds to (100) direction. Note that the needle-like crystal was broken along the longer axis in the sample preparation. The Scale bar is 100 μm.

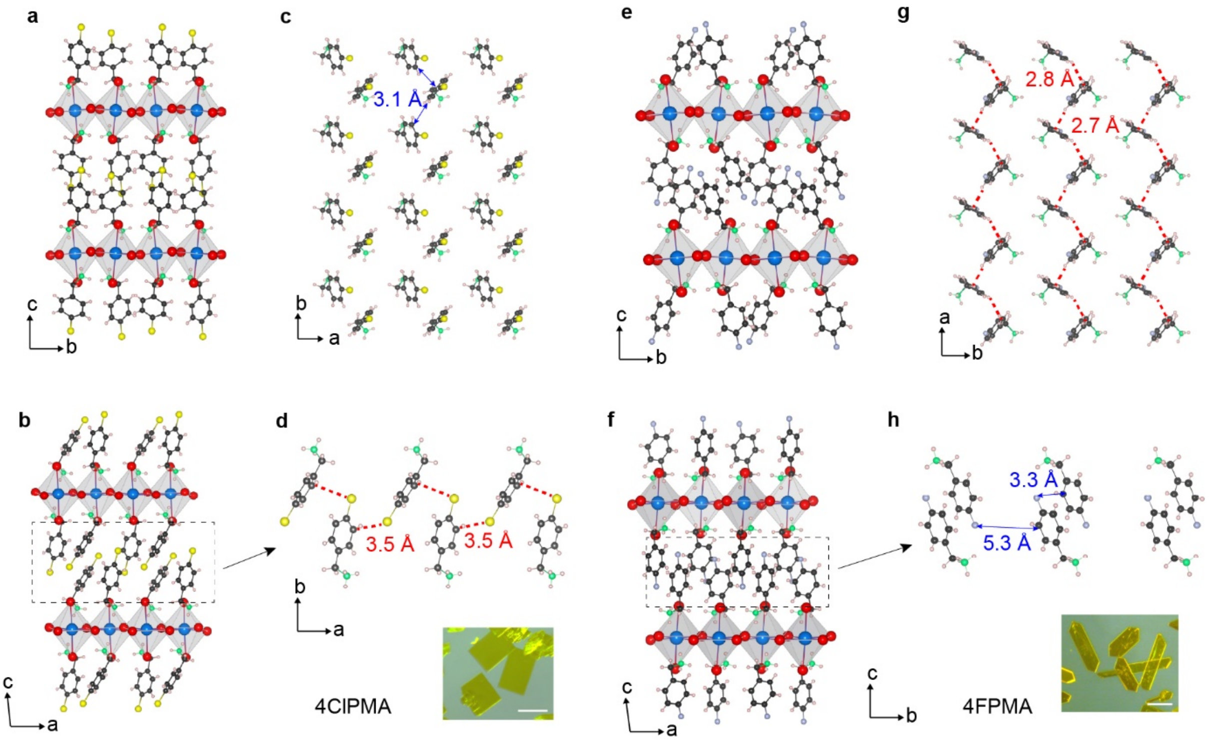

Supplementary Fig. 5 Comparisons of the crystal structures of $(4ClPMA)_2PbI_4$ and $(4FPMA)_2PbI_4$, showing differences in the interlayer and intralayer intermolecular interactions. a, b, Crystal structures of $(4ClPMA)_2PbI_4$ viewed along the *a*-axis (**a**) and along

the *b*-axis (**b**). $(4ClPMA)_2PbI_4$ adopts a space group *P*2_1, with *a* = 8.62 Å, *b* = 8.93 Å, *c* = 15.84 Å, α = γ = 90º, and β = 95.7º. **c**, The arrangement of the $4ClPMA^+$ cations in each layer, exhibiting weaker C-H···π interactions due to the packing configurations disturbed by stronger interlayer halogen-π interactions. **d**, Illustration of the halogen-π interactions in $4ClPMA^+$ cations in the neighboring layers. The scale bar is 100 μm. **e, f**, Crystal structures of $(4FPMA)_2PbI_4$ viewed along the *a*-axis (**e**) and along the *b*-axis (**f**). $(4FPMA)_2PbI_4$ adopts a space group P 21/n, with *a* = 8.70 Å, *b* = 9.24 Å, *c* = 27.53 Å, α = γ = 90º, and β = 97.6º. **g, h**, The arrangement of the $4FPMA^+$ cations in each layer (**g**) and across the neighboring layer (**h**), maintaining the directional C-H···π interactions as observed in $(PMA)_2PbI_4$ due to weaker interlayer F···π interactions. The scale bar is 100 μm. $4ClPMA^+$ = 4-chlorophenylmethylammonium, and $4FPMA^+$ = 4-fluorophenylmethylammonium.

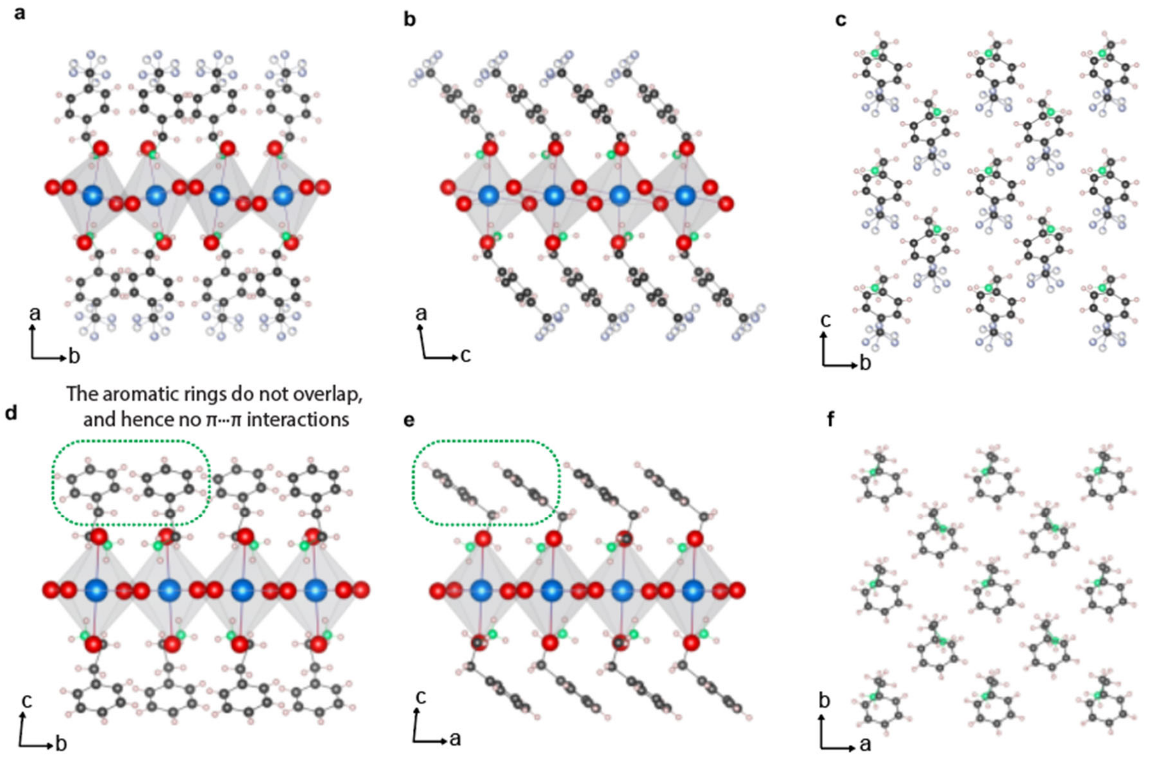

Supplementary Fig. 6 Crystal structures of $(4CF_3PMA)_2PbI_4$ and $(PEA)_2PbI_4$ showing isotropic intermolecular interactions. a, b, Crystal structures of $(4CF_3PMA)_2PbI_4$ viewed along the *c*-axis (**a**) and along the *b*-axis (**b**). $(4CF_3PMA)_2PbI_4$ adopts a space group $P2_1/c$, with a = 18.14 Å, b = 8.42 Å, c = 8.69 Å, α = γ = 90º, and β = 98.9º. **c**, The arrangement of the $4CF_3PMA^+$ cations viewed along the *a*-axis, showing similar intermolecular interactions between $4CF_3PMA^+$ cations along *a*- and *b*-axis. **d, e**, Crystal structures of $(PEA)_2PbI_4$ viewed along the *a*-axis (**d**) and along the *b*-axis (**e**). $(PEA)_2PbI_4$ adopts a space group $P1$, with a = 8.74 Å, b = 8.74 Å, c = 33.02 Å, α = 84.62º, β = 84.63º, and γ = 89.63º. **f**, The arrangement of PEA^+ cations viewed along the *c*-axis, exhibiting similar intermolecular interactions between PEA^+ cations along *a*-axis and *b*-axis. The absence of strong directional anisotropic interactions,

such as C–H···π in or π···π interactions, explains the observed 2D growth in these compounds. PEA^+ = phenylethylammonium, and $4CF_3PMA^+$ = 4-trifluoromethylphenylmethylammonium.

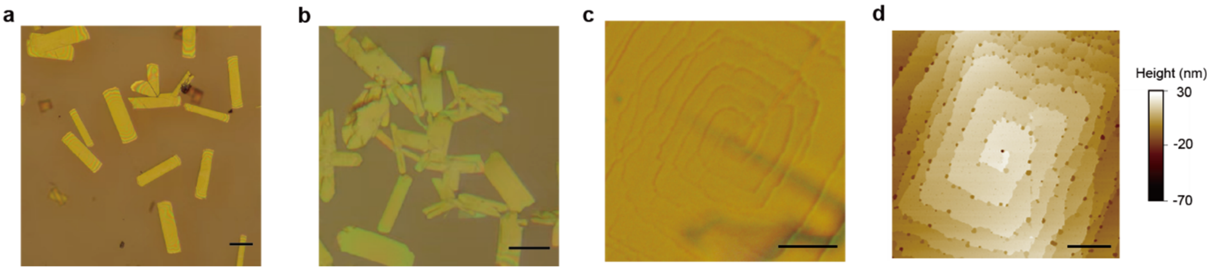

Supplementary Fig 7. The crystal growth of $(BA)_2PbI_4$ and $(HA)_2PbI_4$ showing minor anisotropic growth. **a, b,** Optical images of some $(BA)_2PbI_4$ (**a**) and $(HA)_2PbI_4$ (**b**) crystals with rectangular shape. The scale bars are 25 μm. **c, d,** Optical image (**c**) and AFM image (**d**) showing minor anisotropic screw-dislocation cores in $(BA)_2PbI_4$. The scale bars in c and d are 10 μm and 2 μm, respectively.

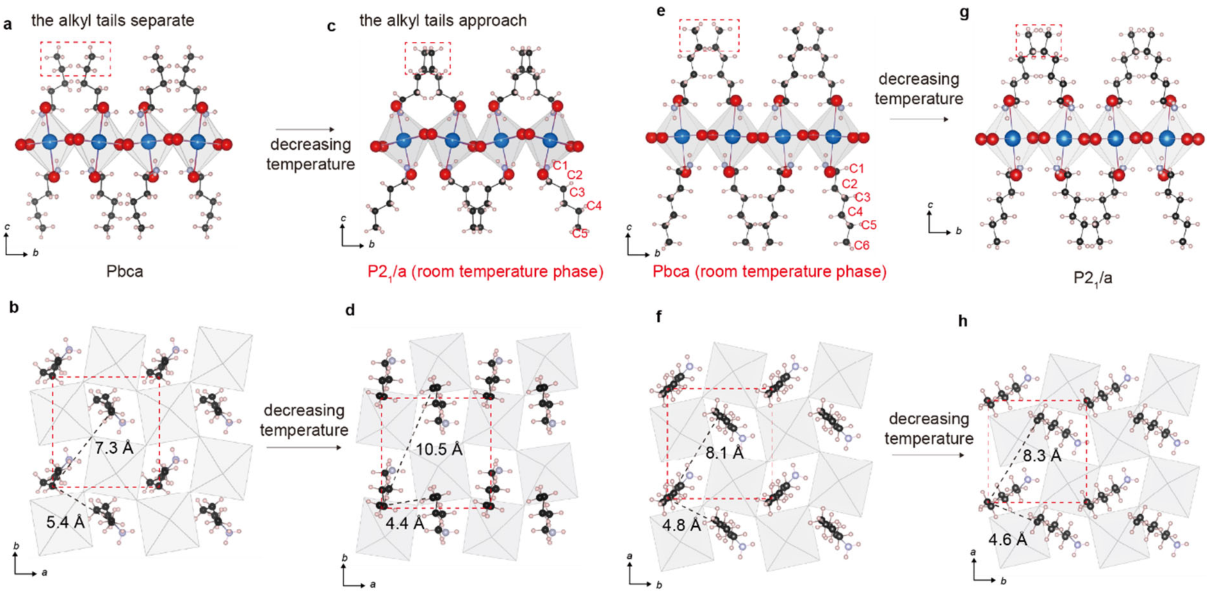

Supplementary Fig 8. Structural phases and the associated intermolecular interactions in $(PA)_2PbI_4$ and $(HA)_2PbI_4$. a, b, Crystal structures of high-temperature phase of $(PA)_2PbI_4$ viewed along the a-axis (**a**) and c-axis (**b**). **c, d,** Crystal structures of low-temperate phase of $(PA)_2PbI_4$ (i.e., room-temperature phase) viewed along the a-axis (**c**) and c-axis (**d**). **e, f,** Crystal structures of high-temperature phase of $(HA)_2PbI_4$ (i.e., room-temperature phase) viewed along the a-axis (**e**) and c-axis (**f**). **g, h,** Crystal structures of low-temperature phase of $(HA)_2PbI_4$ viewed along the a-axis (**g**) and c-axis (**h**).

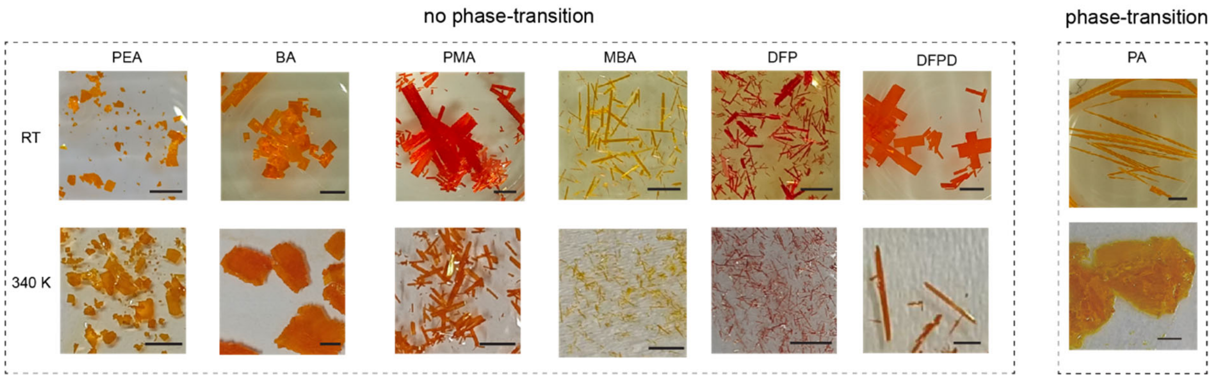

Supplementary Fig 9. Optical images of $(PA)_2PbI_4$ and various other 2D perovskites grown

at room-temperature and 340 K. The scale bars are 2.7 mm. To validate that the morphological transition of $(PA)_2PbI_4$ crystals from plate-like to needle-like at elevated temperatures is driven by the phase transition to a structure with weaker intermolecular interactions, crystal growth behaviors at room temperature and 340 K of various 2D perovskites which do not exhibit phase transitions. Similar growth behaviors at both temperatures are observed. These findings suggest that strong directional intermolecular interactions predominantly influence the crystal shape, rather than the growth temperature

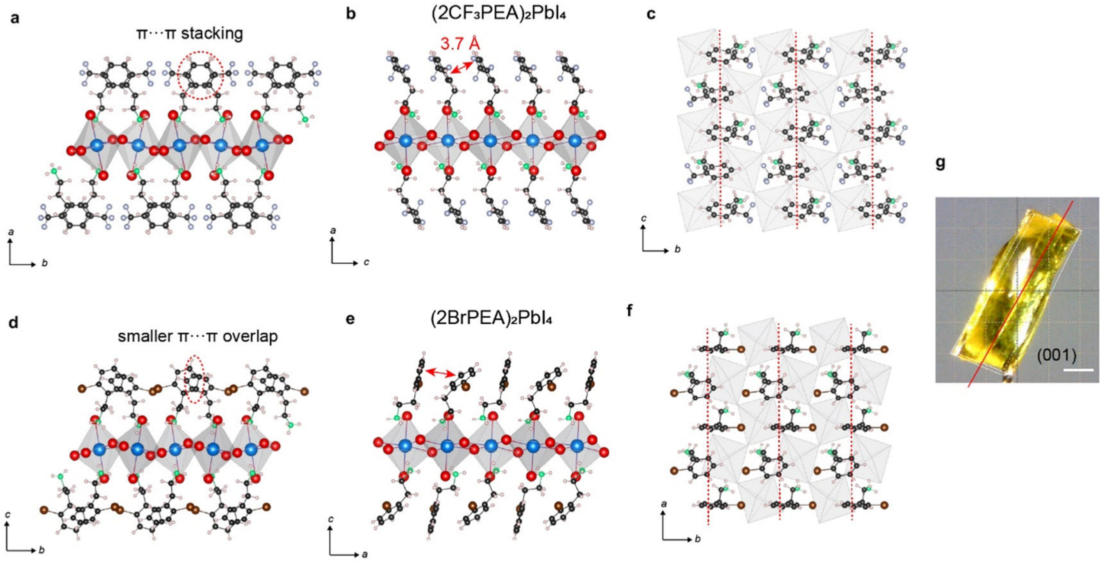

Supplementary Fig 10. Crystal structures of $(2CF_3PEA)_2PbI_4$ and $(2BrPEA)_2PbI_4$ showing the directional intermolecular π···π stacking interactions. a, b, Crystal structures of $(2CF_3PEA)_2PbI_4$ viewed along the *c*-axis (**a**) and along the *b*-axis (**b**). $(2CF_3PEA)_2PbI_4$ adopts a space group *P21/c*, with a = 19.37 Å, b = 8.53 Å, c = 8.66 Å, and $\alpha = \gamma = 90^o$, β =91.8 º. **c**, The arrangement of the $2CF_3PEA^+$ cations and inorganic framework viewed along the *a*-axis, showing directional intermolecular π···π stacking interactions along the *c*-axis. **d, e**, Crystal structures of $(2BrPEA)_2PbI_4$ viewed along the *a*-axis (**d**) and along the *b*-axis (**e**).

$(2BrPEA)_2PbI_4$ adopts a space group *P 21*, with *a* = 8.89 Å, *b* = 8.16 Å, *c* = 17.99 Å, and α = γ = 90º, β =94.8 º. **f**, The arrangement of the $2BrPEA^+$ cations viewed along the *c*-axis, showing directional intermolecular π···π stacking interactions along the *a*-axis. **g**, Single-crystal X-ray diffraction measurements revealed that the longer axis of $(2CF_3PEA)_2PbI_4$ is consistent with the direction of intermolecular interactions. The scale bar is 100 μm.

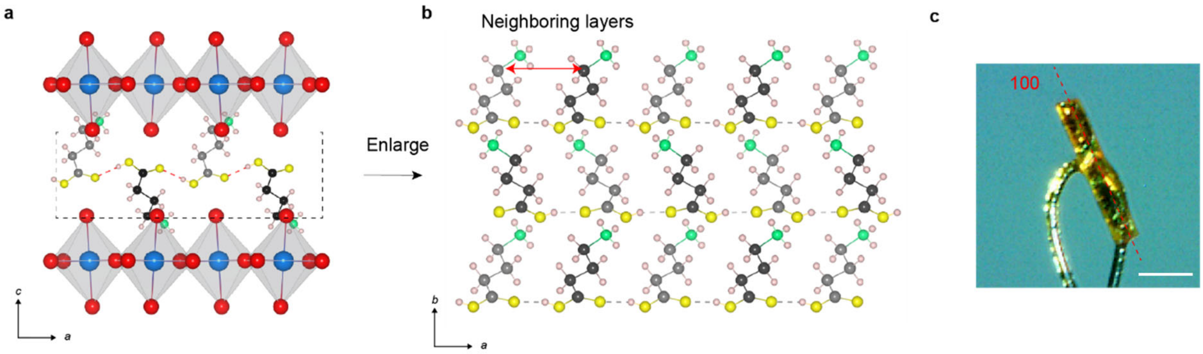

Supplementary Fig. 11. Crystal structures of $(ABA)_2PbI_4$ showing the formation of directional interlayer hydrogen bond in the ABA^+ cations and single-crystal X-ray diffraction measurement on $(ABA)_2PbI_4$. a, Crystal structure of $(ABA)_2PbI_4$ viewed along the *b*-axis. $(ABA)_2PbI_4$ adopts a space group *P*bca, with *a* = 8.91 Å, *b* = 9.28 Å, *c* = 24.35Å, and α = β = γ = 90º. **b**, The arrangement of the ABA^+ cations in the two adjacent layers, showing interlayer directional hydrogen bonds. **c**, Optical image of a $(ABA)_2PbI_4$ crystal mounted on the diffractor, showing the longer axis which corresponds to (100) direction. The scale bar is 100 μm.

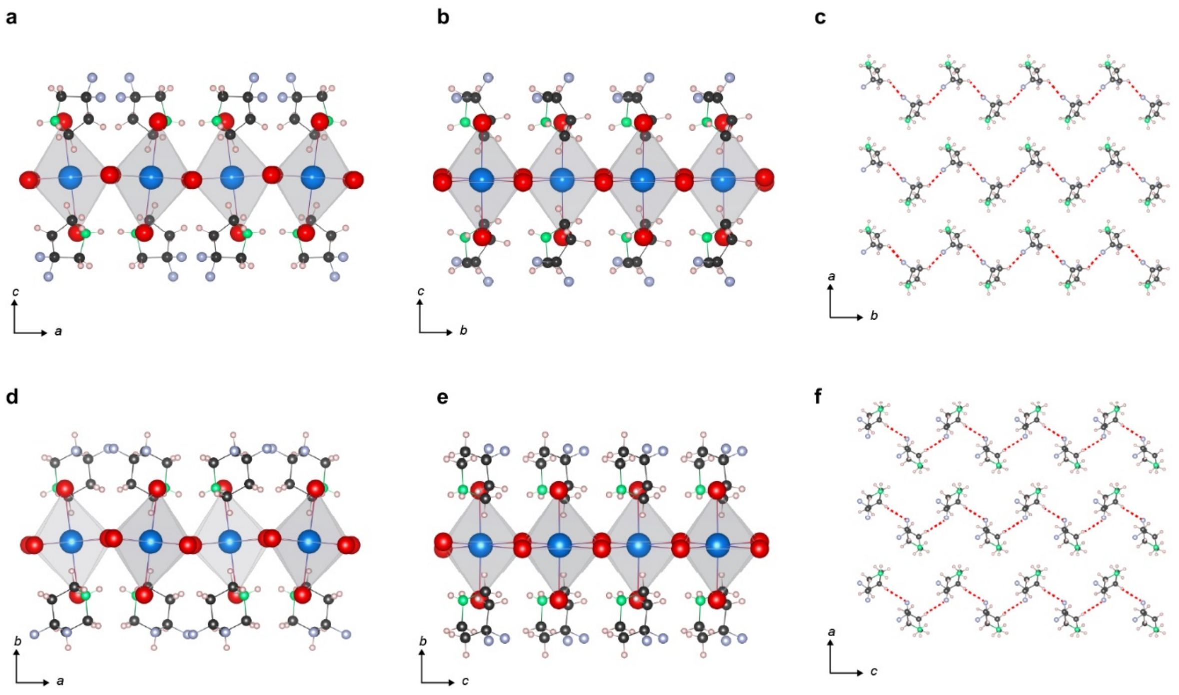

Supplementary Fig. 12. Crystal structures of $(DFP)_2PbI_4$ and $(DFPD)_2PbI_4$ showing the directional intermolecular F···H interactions. a, b, Crystal structures of $(DFP)_2PbI_4$ viewed along the *b*-axis (**a**) and along the *a*-axis (**b**). $(DFP)_2PbI_4$ adopts a space group *P*bcn, with *a* = 9.29 Å, *b* = 8.98 Å, *c* = 23.32 Å, and α = β = γ = 90º. **c**, The arrangement of the DFP^+ cations viewed along the *c*-axis, showing directional intermolecular F···H interactions along the *b*-axis. **d, e**, Crystal structures of $(DFPD)_2PbI_4$ viewed along the *c*-axis (**d**) and along the *a*-axis (**e**). $(DFPD)_2PbI_4$ adopts a space group *A*ea2, with *a* = 9.36 Å, *b* = 25.36 Å, *c* = 9.01 Å, and α = β = γ = 90º. **f**, The arrangement of the $DFPD^+$ cations viewed along the *b*-axis, showing directional intermolecular F···H interactions along the *c*-axis. Single-crystal X-ray diffraction measurements revealed that the longer axis is consistent with the direction of intermolecular interactions.

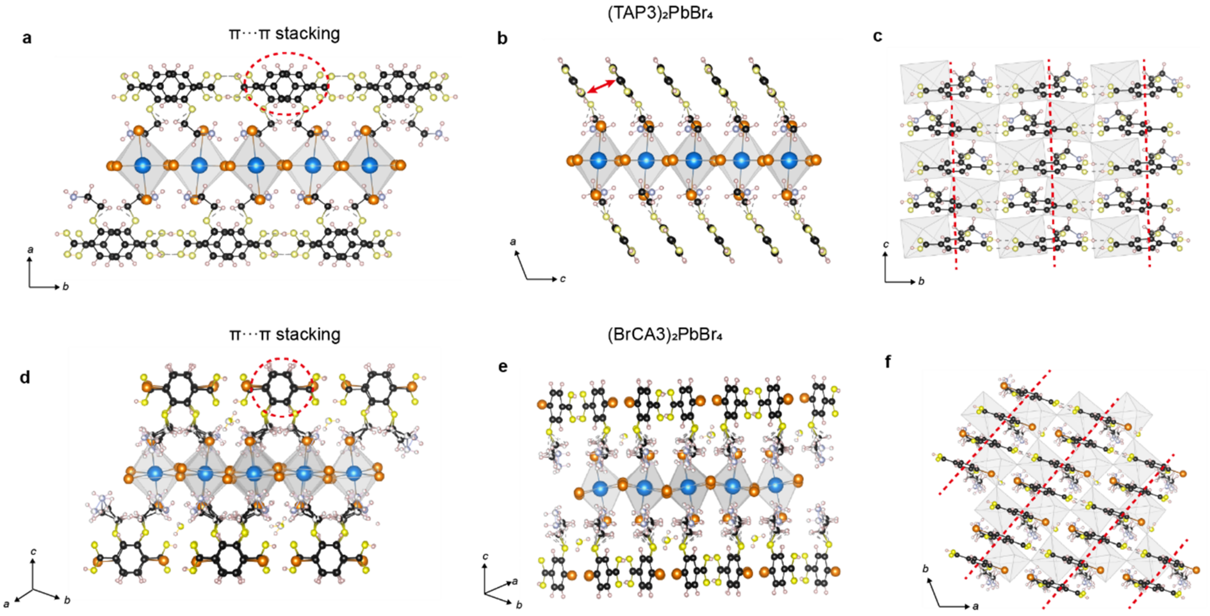

Supplementary Fig. 13. Crystal structures of $(TPA3)_2PbBr_4$ and $(BCA3)_2PbBr_4$ showing the directional intermolecular π···π stacking interactions. a, b, Crystal structures of $(TPA3)_2PbBr_4$ viewed along the *c*-axis (**a**) and along the *b*-axis (**b**). $(TPA3)_2PbBr_4$ adopts a space group $P2_1/c$, with *a* =18.25 Å, *b* = 9.54 Å, *c* = 7.90 Å, and α = γ = 90º, β =101.4 º. **c**, The arrangement of the $TPA3^+$ cations and inorganic framework viewed along the out-of-plane direction, showing directional intermolecular π···π stacking interactions along the *c*-axis. **d, e**, Crystal structures of $(BrCA3)_2PbBr_4$ viewed along the (110) direction (**d**) and along the (-110) direction (**e**). $(BrCA3)_2PbBr_4$ adopts a space group *P 1*, with *a* = 11.81 Å, *b* = 11.89 Å, *c* = 20.57 Å, and α =97.4 º, β =97.2 º, γ = 96.8º. **f**, The arrangement of the $BrCA3^+$ cations viewed along the *c*-axis, showing directional intermolecular π···π stacking interactions along the (110) direction. Single-crystal X-ray diffraction measurements revealed that the longer axis is consistent with the direction of intermolecular interactions.

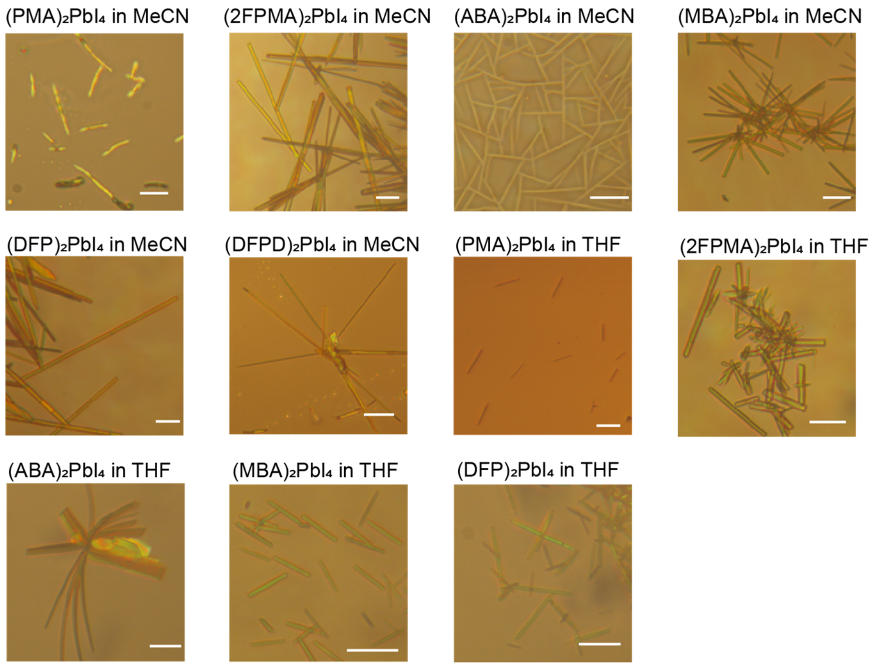

Supplementary Fig. 14 Optical images of 2D perovskite nanowires grown in organic solvents. In certain structures, branching was observed, which arises from multiple nucleation sites during crystal growth. This phenomenon is promoted by the rapid or abrupt evaporation of the organic solvent, leading to a higher degree of supersaturation. It should also be noted that the nanowire growth in organic solvents has not been carefully optimized. By reducing the evaporation rate of the organic solvent, branching can be mitigated. All scale bars are 25 μm. MeCN = acetonitrile, THF = tetrahydrofuran.

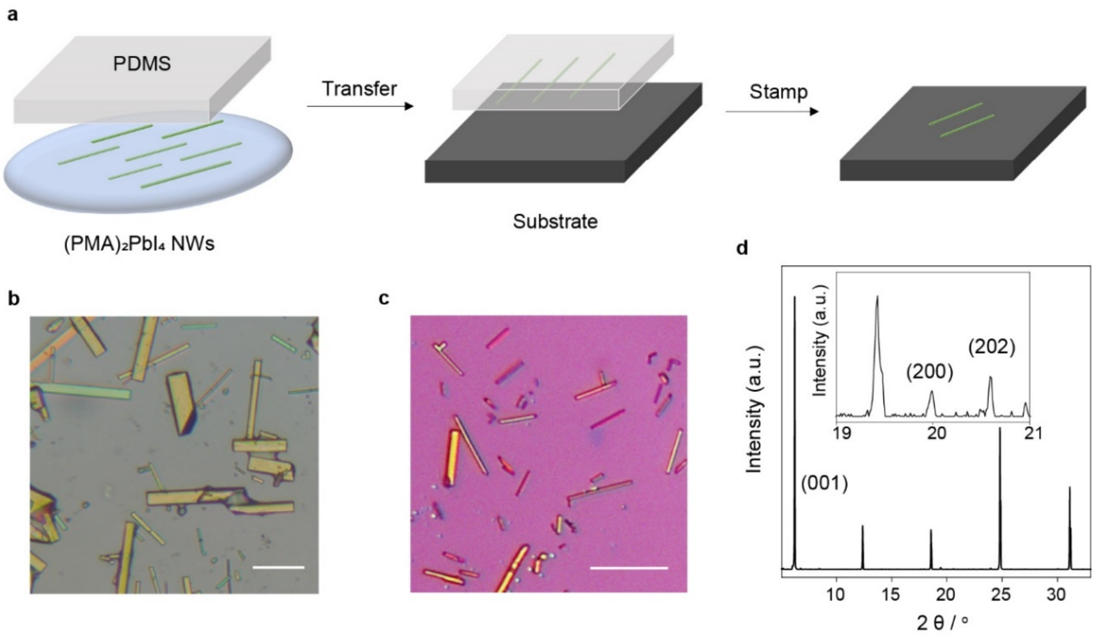

Supplementary Fig. 15. Transfer the as-grown nanowires from the solution to arbitrary substrates and powder X-ray diffraction characterization of the nanowires. **a**, Schematic illustration of the method for picking up and transferring 2D perovskite nanowires. **b, c**, Optical images of $(PMA)_2PbI_4$ nanowires, which were picked up by PDMS (**b**) and transferred onto Si/SiO_2 substrate (**c**). **d**, Powder X-ray diffraction pattern of transferred $(PMA)_2PbI_4$ nanowires. All scale bars are 25 μm. PMA^+ = phenylmethylammonium.

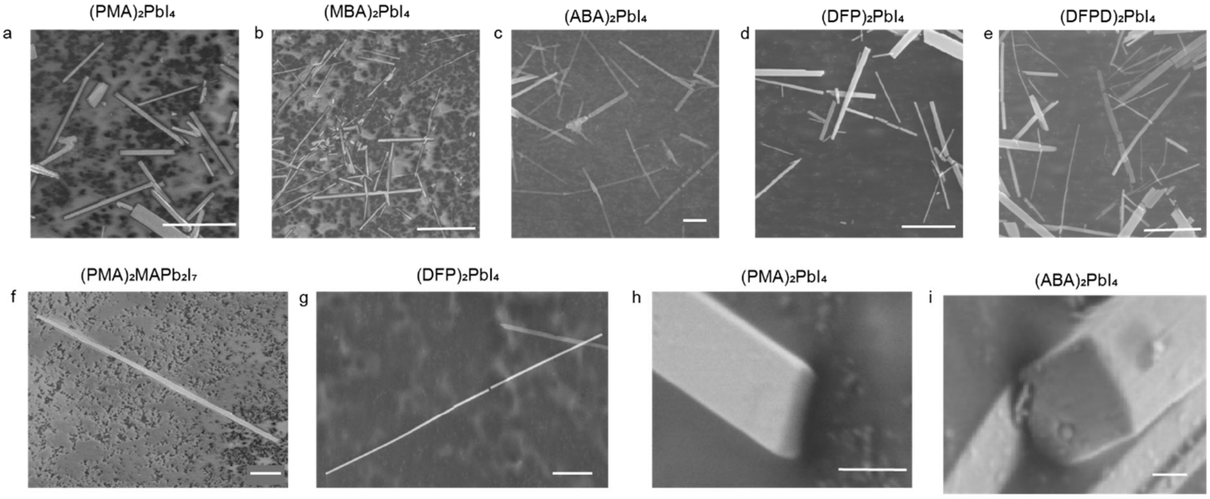

Supplementary Fig. 16. SEM images of several representative NWs. a-g, SEM images of $(PMA)_2PbI_4$ (**a**), $(MBA)_2PbI_4$ (**b**), $(ABA)_2PbI_4$ (**c**), $(DFP)_2PbI_4$ (**d** and **g**), $(DFPD)_2PbI_4$ (**e**) and $(PMA)_2MAPb_2I_7$ (**f**) nanowires. The scale bars are 10 μm. **h, i,** SEM images of cross sections of $(PMA)_2PbI_4$ (**h**) and $(ABA)_2PbI_4$ (**i**) nanowires. The scale bars are 1 μm.

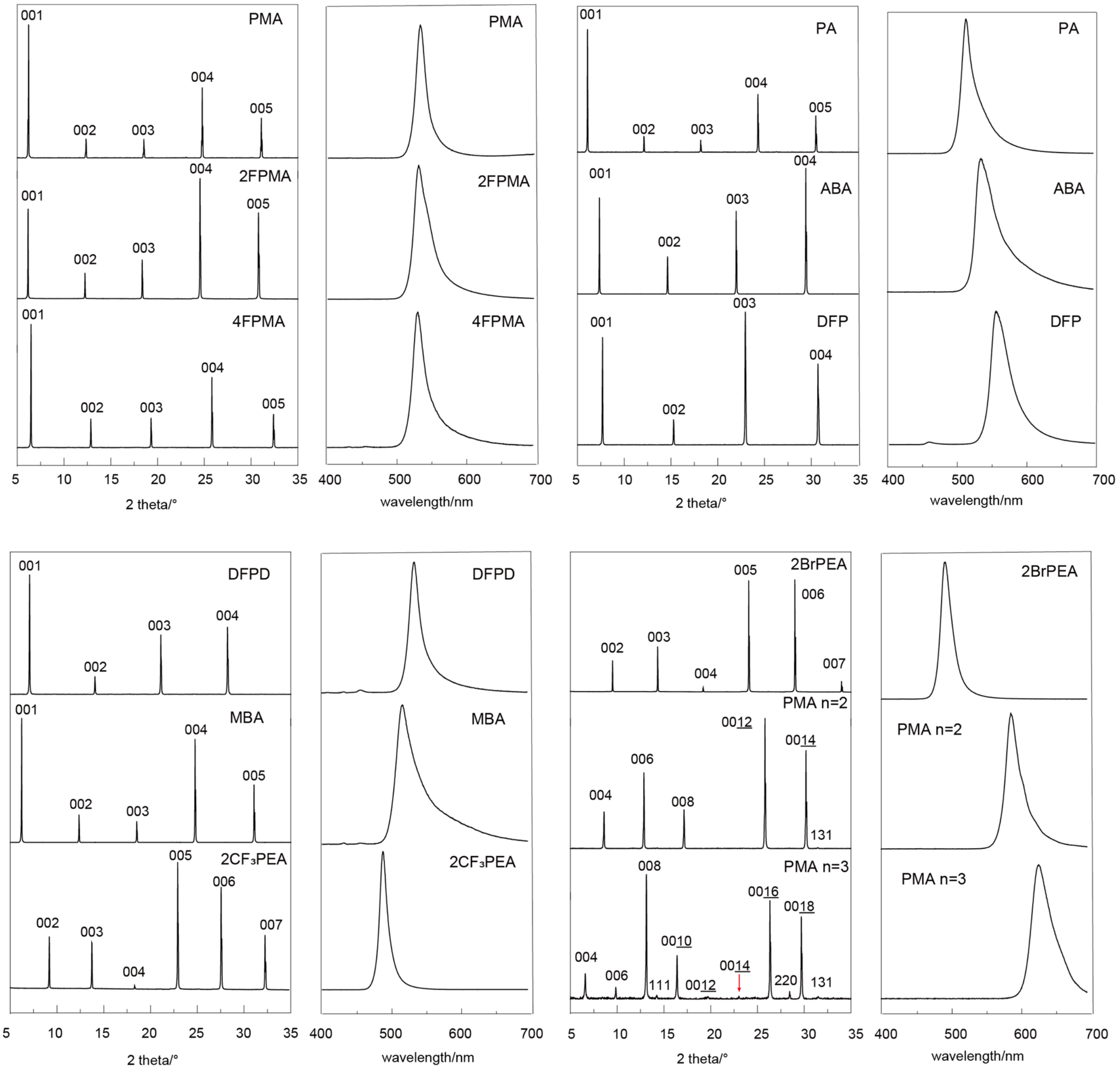

Supplementary Fig. 17. Powder X-ray diffraction patterns and PL spectra of various lead iodide perovskite nanowires confirming the 2D perovskite phases.

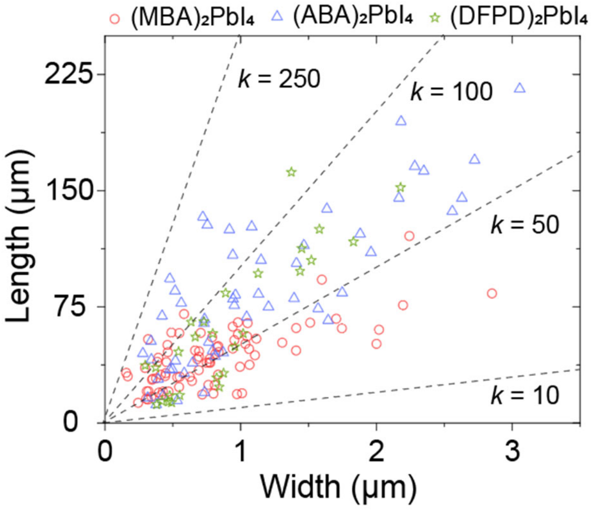

Supplementary Fig. 18. Statistics of the width and length distributions of $(MBA)_2PbI_4$, $(ABA)_2PbI_4$, and $(DFPD)_2PbI_4$ NWs

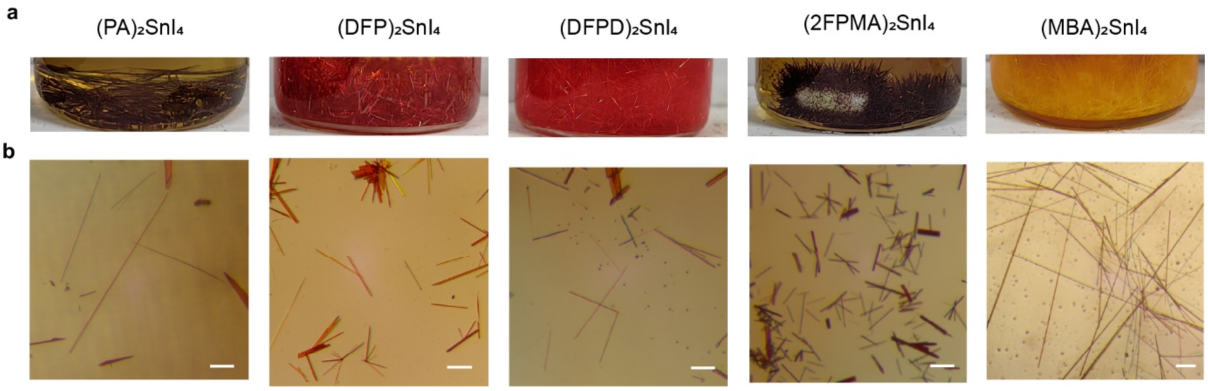

Supplementary Fig. 19. Needle-like crystals and nanowires of 2D tin iodide perovskites. **a**, Optical images of various $(LA)_2SnI_4$ crystals that grow into needles. **b**, Optical images of the corresponding $(LA)_2SnI_4$. Scale bars in $(PA)_2SnI_4$, $(DFP)_2SnI_4$, $(DFPD)_2SnI_4$, and $(2FPMA)_2SnI_4$ are 25 μm. Scale bar in $(MBA)_2SnI_4$ is 50 μm.

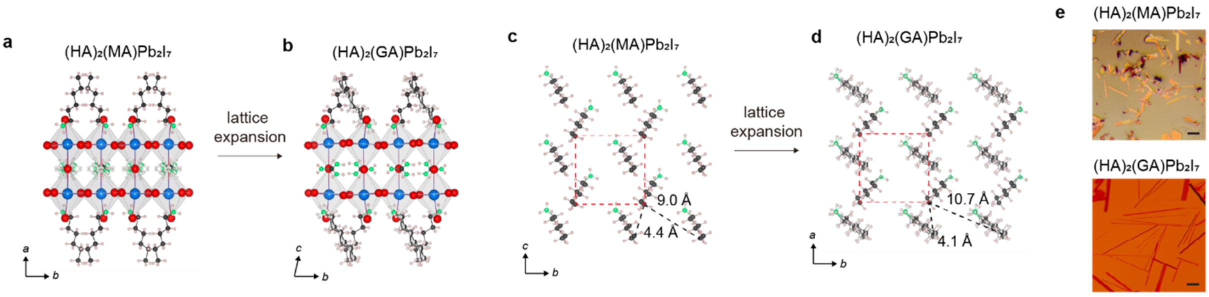

Supplementary Fig. 20. Comparisons of the crystal structures and anisotropic Van der Waals interactions of the HA$^+$ cations in $(HA)_2(MA)Pb_2I_7$ and $(HA)_2(GA)Pb_2I_7$. a, b, Crystal structures of $(HA)_2(MA)Pb_2I_7$ (**a**) and $(HA)_2(GA)Pb_2I_7$ (**b**) viewed along the in-plane direction. **c, d**, The arrangement of the HA$^+$ cations in $(HA)_2(MA)Pb_2I_7$ (**c**) and $(HA)_2(GA)Pb_2I_7$ (**d**) viewed along the out-of-plane direction. **e**, Optical images of $(HA)_2(MA)Pb_2I_7$ with elongated-shape and $(HA)_2(GA)Pb_2I_7$ NWs with larger aspect ratios due to stronger intermolecular interaction anisotropy. Scale bars are 25 µm. $(HA)_2(MA)Pb_2I_7$ adopts a space group *C2/c*, with a = 45.146 Å, b = 8.814 Å, c = 8.695 Å, $(HA)_2(GA)Pb_2I_7$ adopts a space group *P*-1, with a = 8.8195 Å, b = 9.0300 Å, c = 21.699 Å, and α = 79.965°, β = 87.341°, γ = 89.986°. In $(HA)_2(MA)Pb_2I_7$, the tails of the HA cations exhibit off-centering displacement due to anisotropic intermolecular interactions. When the inorganic lattice is expanded by incorporating larger GA$^+$ cations, the off-centering displacement of the HA$^+$ cation tails is further enhanced, as evidenced by the carbon–carbon distances reported in Table S3. As a result, $(HA)_2(GA)Pb_2I_7$ exhibits greater anisotropy in the inorganic lattice compared to $(HA)_2(MA)Pb_2I_7$, as also reflected by the difference in the in-plane lattice parameters.

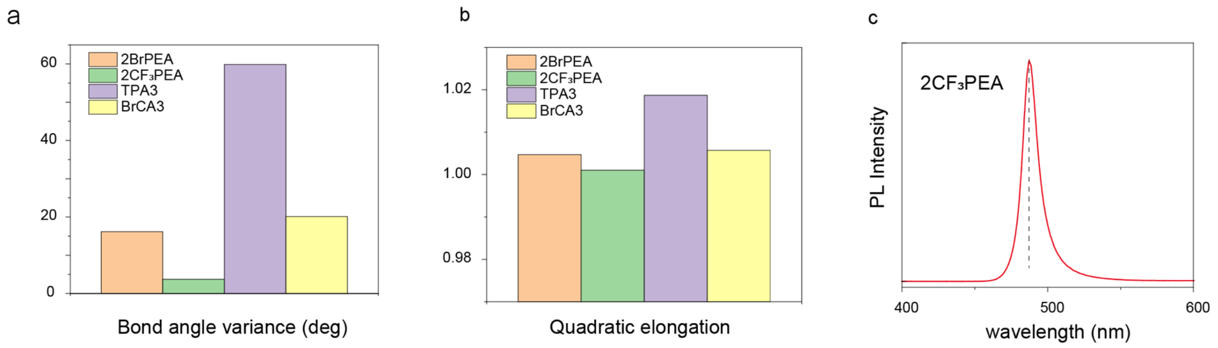

Supplementary Fig. 21. Comparisons of octahedral distortions and PL properties of between $(BrCA3)_2PbI_4$ and our samples such as $(2BrPEA)_2PbI_4$ and $(2CF_3PEA)_2PbI_4$. The octahedral distortions were quantified using quadratic elongation ($\langle\lambda\rangle$) and bond angle variance (σ_θ^2). $\langle\lambda\rangle = \sum_{i=1}^{6}(l_i/l_0)^2/6$, where l_i is the individual Pb–I bond length, l_0 is the average Pb–I bond length, and $\sigma_\theta^2 = \sum_{i=1}^{12}(\theta_i - 90°)^2/11$, with θ_i being I–Pb–I bond angle of neighboring iodides. Higher values of $\langle\lambda\rangle$ and σ_θ^2 indicate a more distorted octahedron. The PL asymmetric factor is defined as the ratio of integrated PL intensity on the low-energy side relative to the high-energy side, with a higher value indicating stronger exciton-photon coupling.

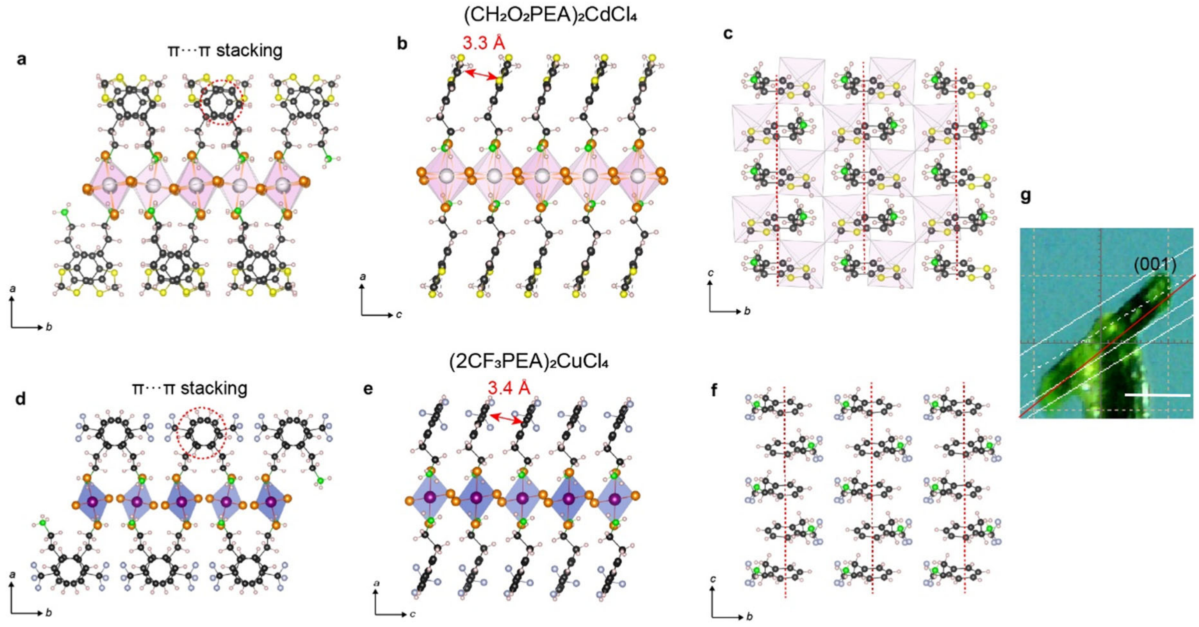

Supplementary Fig. 22. Crystal structures of $(CH_2O_2PEA)_2CdCl_4$ and $(2CF_3PEA)_2CuCl_4$ showing the directional intermolecular π···π stacking interactions. a, b, Crystal structures of $(CH_2O_2PEA)_2CdCl_4$ viewed along the *c*-axis (**a**) and along the *b*-axis (**b**). $(CH_2O_2PEA)_2CdCl_4$ adopts a space group *P21/c*, with *a* = 20.37 Å, *b* = 7.43 Å, *c* = 7.49 Å, and α = γ = 90º, β =91.2 º. **c**, The arrangement of the $CH_2O_2PEA^+$ cations and inorganic framework viewed along the *a*-axis, showing directional intermolecular π···π stacking interactions along the *c*-axis. **d, e**, Crystal structures of $(2CF_3PEA)_2CuCl_4$ viewed along the *c*-axis (**d**) and along the *b*-axis (**e**). $(2CF_3PEA)_2CuCl_4$ adopts a space group P21/c, with *a* = 18.12 Å, *b* = 8.25 Å, *c* = 7.67 Å, and α = γ = 90º, β =90.6 º. **f**, The arrangement of the $2CF_3PEA^+$ cations viewed along the *a*-axis, showing directional intermolecular π···π stacking interactions along the *c*-axis. **g**, Single-crystal X-ray diffraction measurements revealed that the longer axis of

$(2CF_3PEA)_2CuCl_4$ is consistent with the direction of intermolecular interactions. The scale bar is 100 μm.

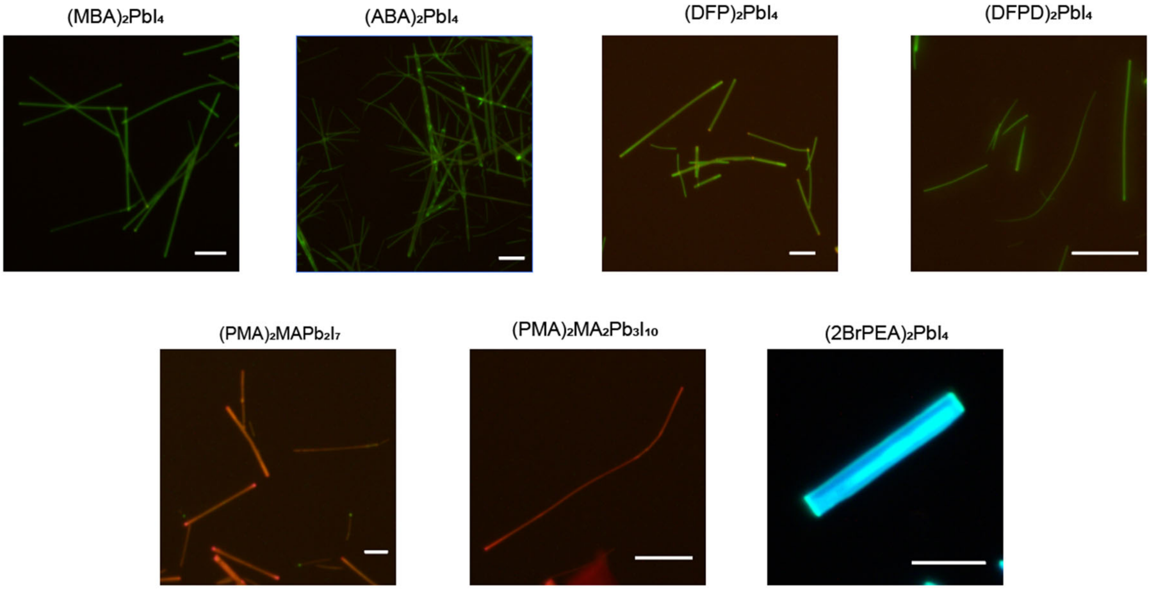

Supplementary Fig. 23. PL images of several representative NWs. The samples were illuminated using a mercury lamp, equipped with an excitation bandpass filter centered at 400 ± 5 nm. The emitted light was detected by a color camera, after passing through a 425 nm long-pass emission filter. All scale bar 10 μm.

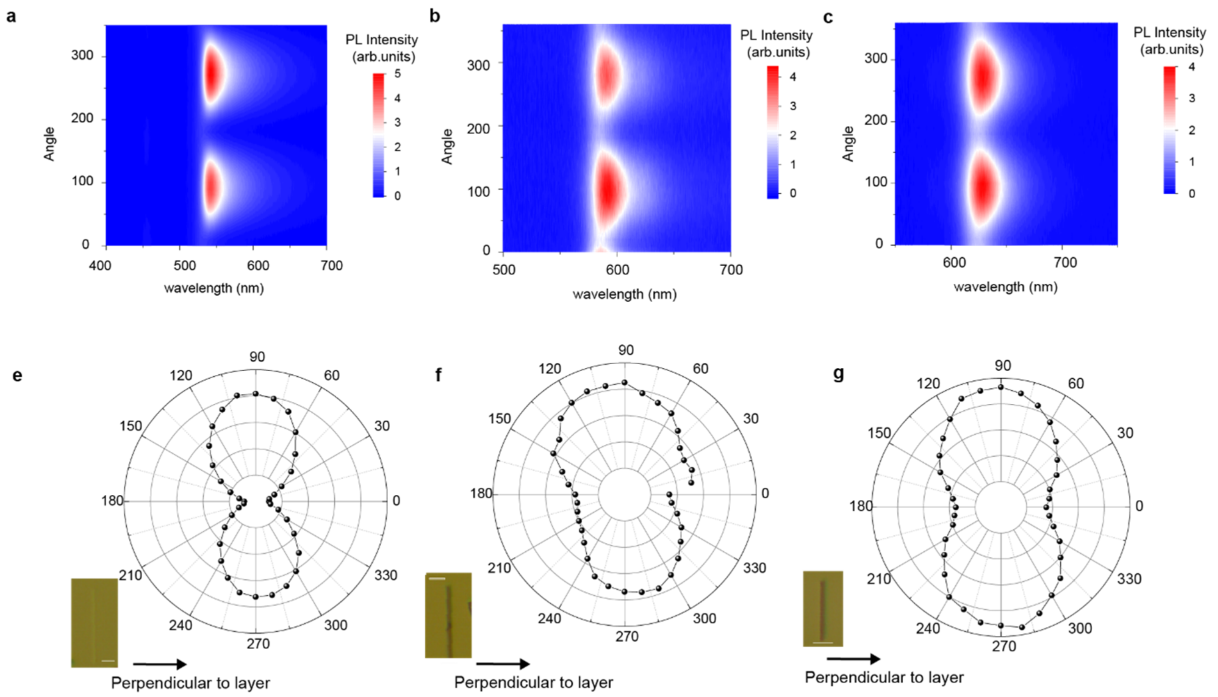

Supplementary Fig. 24. Anisotropic PL emission of several representative NWs. a-c, 2D color plot of the polarization resolved PL spectra of three representative NWs $(DFPD)_2PbI_4$ (n = 1, **a**), $(PMA)_2(MA)Pb_2I_7$ (n = 2, **b**), and $(PMA)_2(MA)_2Pb_3I_{10}$ (n = 3, **c**). **e-f,** Polar plots of the PL intensity with different emission polarizations for the three NWs $(DFPD)_2PbI_4$ (**d**), $(PMA)_2(MA)Pb_2I_7$ (**e**), and $(PMA)_2(MA)_2Pb_3I_{10}$ (**f**), showing the emission polarization along the NW direction. The NWs were rotated such that the out-of-plane direction was perpendicular to the substrates. Insets show the alignment of nanowires, the scale bars are 10 μm. The degree of polarization (DOP) is given by $\text{DOP} = (I_{max} - I_{min})/(I_{max} + I_{min})$, where I_{max} is the maximum integrated PL intensity and I_{min} is the minimal integrated PL intensity.

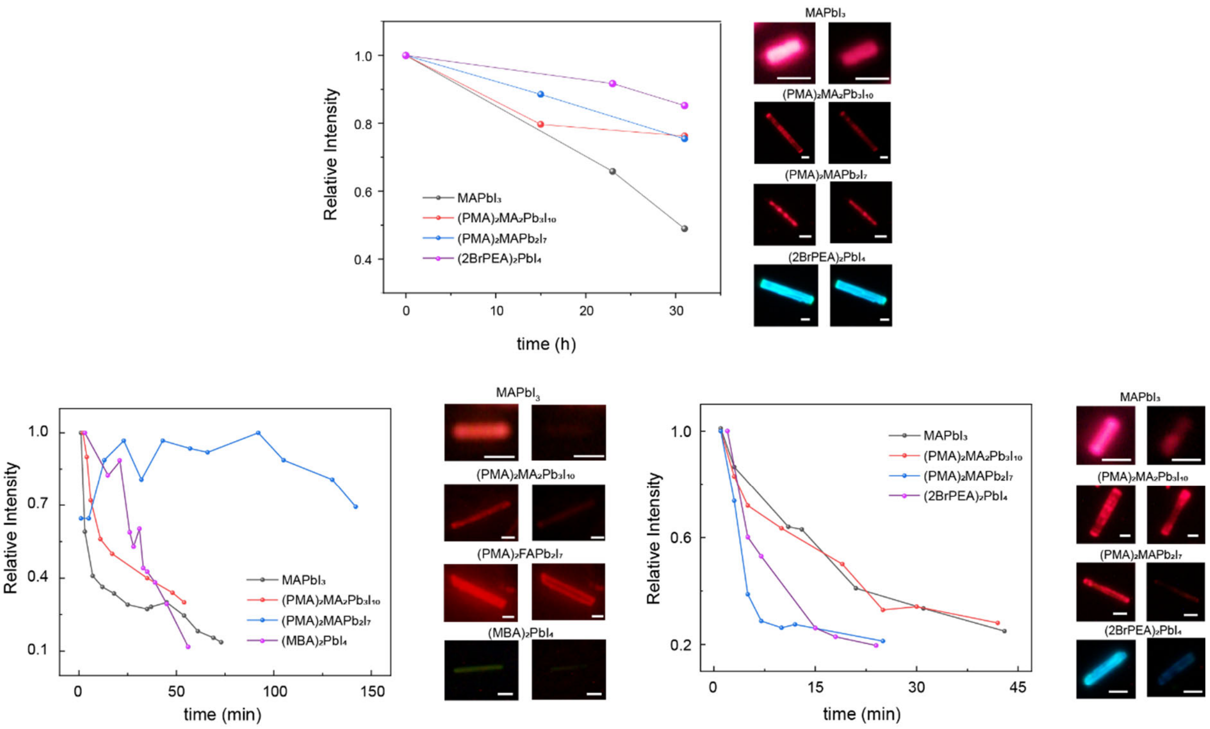

Supplementary Fig. 25. Comparisons of air stability, thermal stability, and photostability between 3D perovskite NWs and 2D perovskite NWs. The left panel shows the plot of PL intensity versus time. The right panel shows the PL image of the samples before and after degradation. All scale bars are 5 μm. **a,** Air stability under room temperature and humidity 76%. **b,** Thermal stability under 60 ºC and humidity 43% in air. **c,** Photostability under 400 ± 5 nm light illumination from a mercury lamp and humidity 43% in air. The light power density is 0.014 W/cm^2.

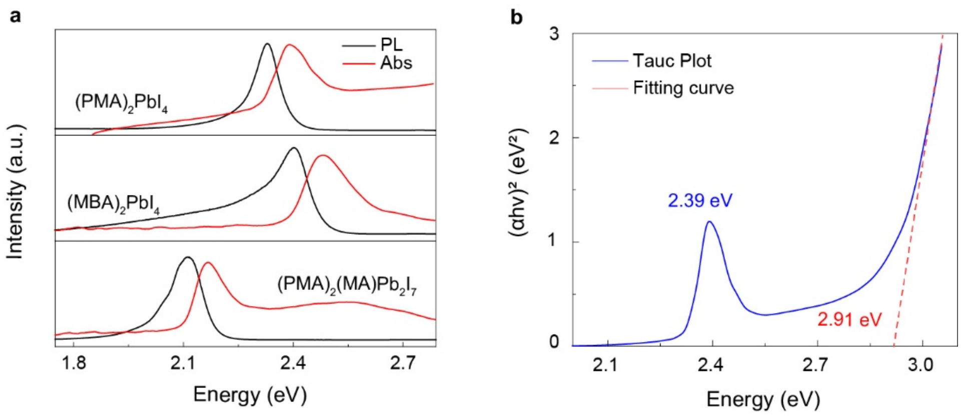

Supplementary Fig. 26. Optical characterizations of various 2D perovskite nanowires. a, Photoluminescence (PL) and absorption spectra of $(PMA)_2PbI_4$, $(MBA)_2PbI_4$, and $(PMA)_2(MA)Pb_2I_7$. **b**, Fitting curve for the exciton binding energy of $(PMA)_2PbI_4$ using Tauc plot.

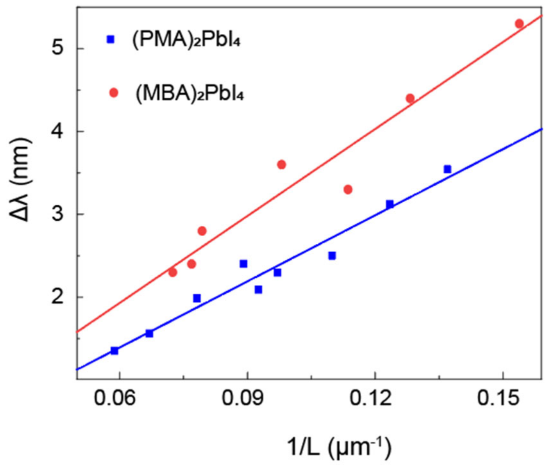

Supplementary Fig. 27. Confirmation of the Fabry-Perot cavity modes. Fitting curves between the mode spacing and the inverse length of the NWs for $(PMA)_2PbI_4$ and $(MBA)_2PbI_4$. PMA^+ = phenylmethylammonium, and MBA^+ = methylbenzylammonium.

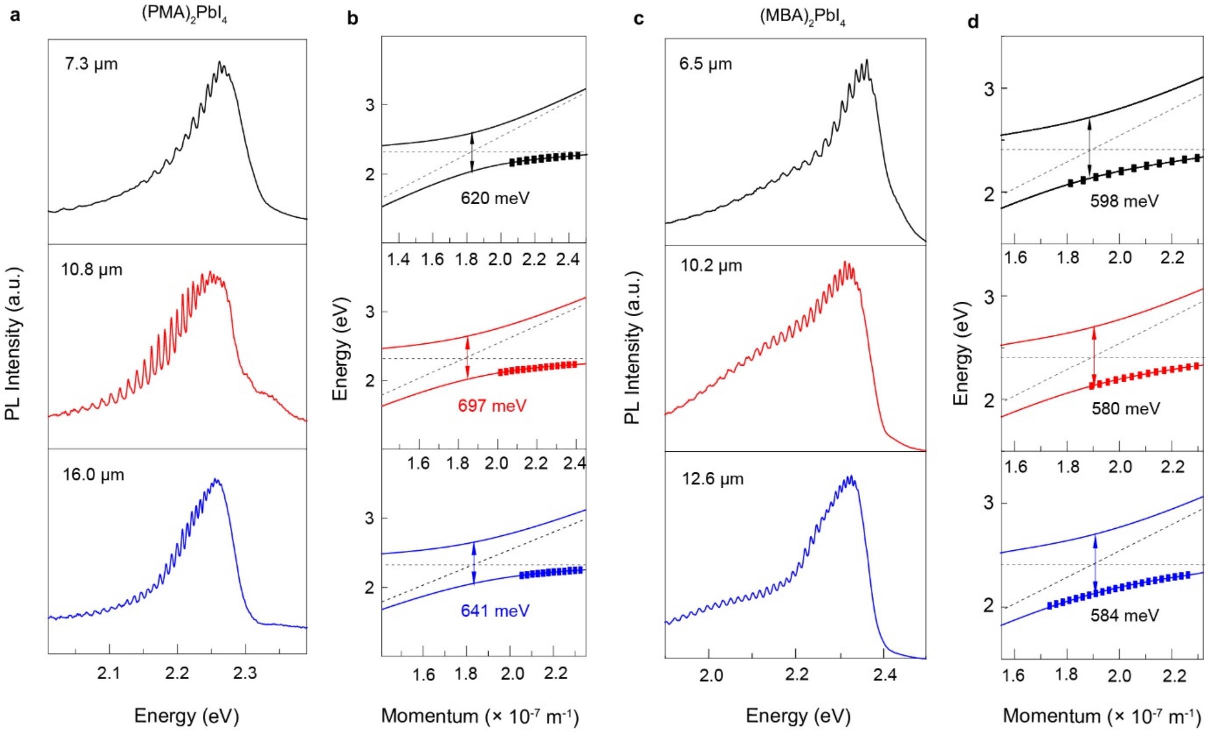

Supplementary Fig. 28. Analysis of exciton-photon coupling in 2D perovskite nanowires with different lengths. a, c, Spatially resolved photoluminescence (PL) spectra collected from the end of several $(PMA)_2PbI_4$ (**a**) and $(MBA)_2PbI_4$ (**c**) NWs with different lengths. **b, d**, Dispersion curves for the corresponding NWs. The dots are the experimental data, the solid lines are the fitting dispersion curves of the exciton-polaritons, and dashed lines are the dispersion curves of the uncoupled cavity photons or excitons. PMA^+ = phenylmethylammonium, and MBA^+ = methylbenzylammonium.

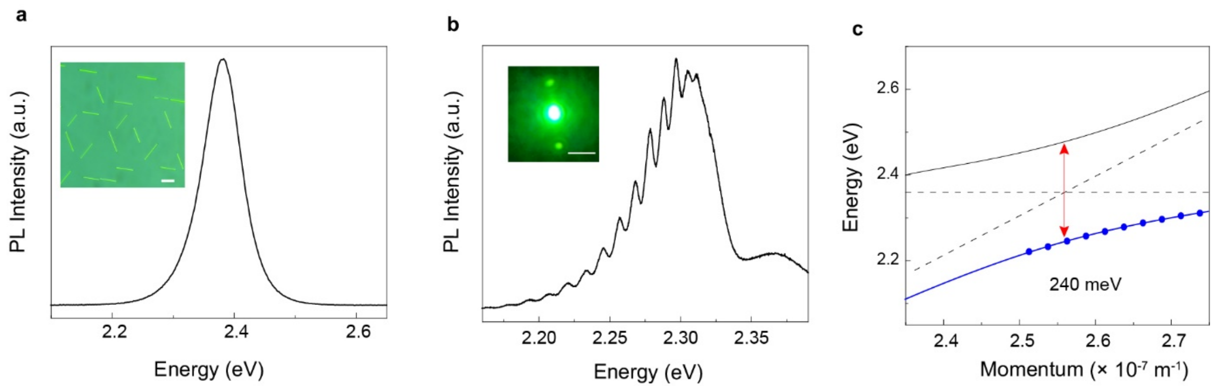

Supplementary Fig. 29. Analysis of exciton-photon coupling in 3D perovskite nanowires. **a**, Photoluminescence (PL) spectrum of a $CsPbBr_3$ NW. Inset shows optical image of $CsPbBr_3$ NWs grown on mica substrate. Scale bar is 6 μm. **b**, Spatially resolved PL spectrum collected from the end of a $CsPbBr_3$ NW. **c**, Dispersion curves for the $CsPbBr_3$ NW. The dots are the experimental data, the solid lines are the fitting dispersion curves of the exciton-polaritons, and dashed lines are the dispersion curves of the uncoupled cavity photons or excitons.

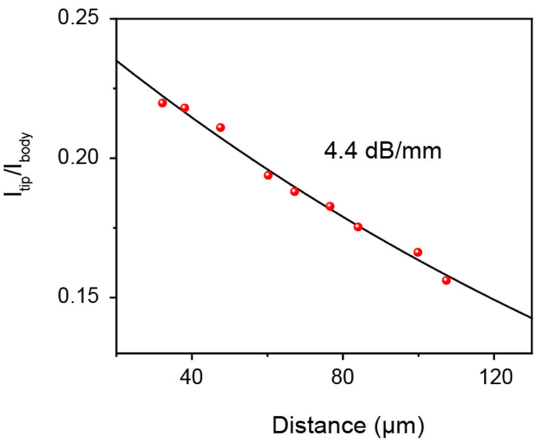

Supplementary Fig. 30. **The tip emission intensity relative to the intensity at the excitation spot as function of the propagation distance for a representative NW.** The optical loss coefficient is measured to be 4.4 dB/mm.

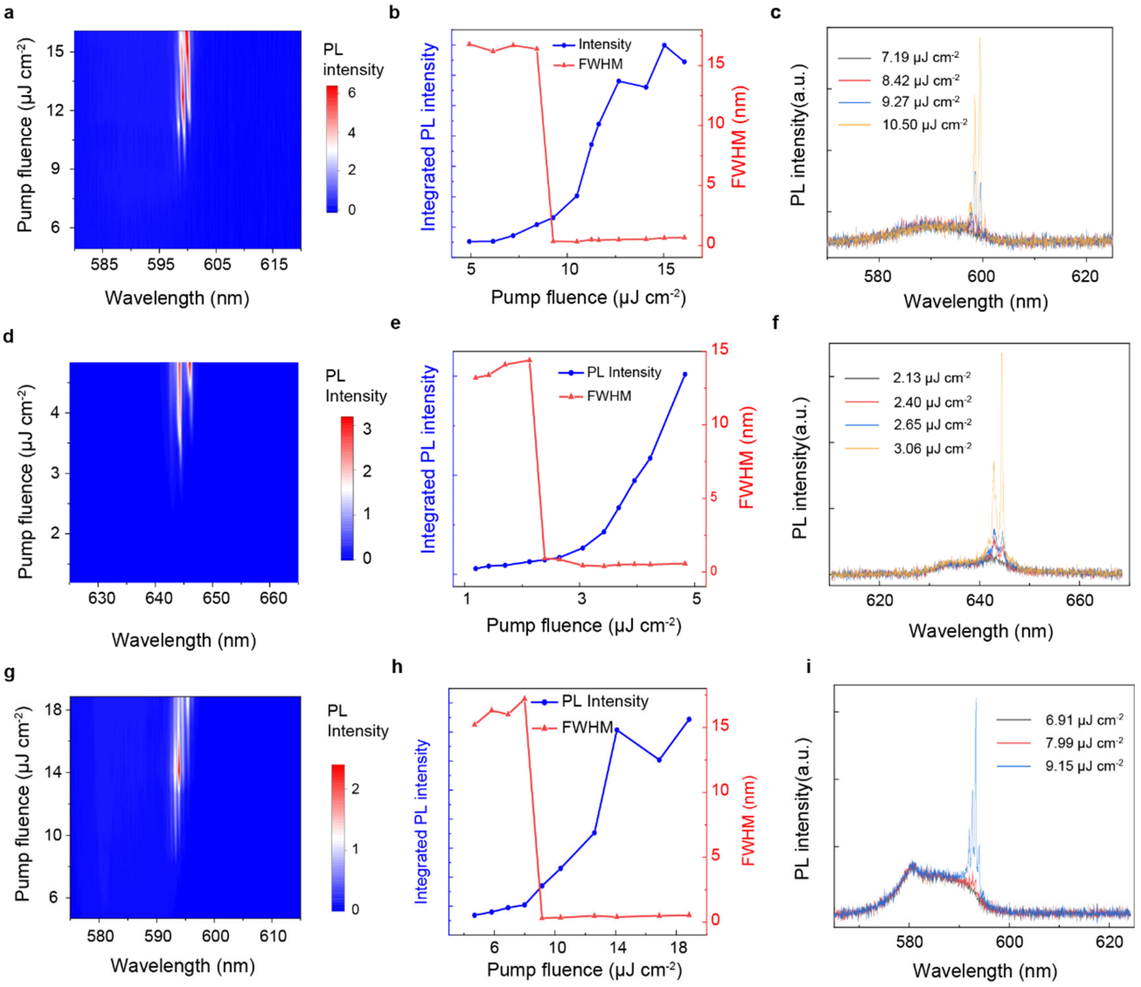

Supplementary Fig. 31. Lasing studies of various 2D perovskite nanowires. Lasing characterizations of $(PMA)_2(MA)Pb_2I_7$ (**a-c**), $(PMA)_2(MA)_2Pb_3I_{10}$ (**d-f**), and $(HA)_2(GA)Pb_2I_7$ (**g-i**) NWs. 2D color plot of the emission spectra of $(PMA)_2(MA)Pb_2I_7$ (**a**), $(PMA)_2(MA)_2Pb_3I_{10}$ (**d**), and $(HA)_2(GA)Pb_2I_7$ (**g**) NWs under different pump fluences. Integrated PL emission intensity and FWHM of the emission peaks versus the pump fluences for $(PMA)_2(MA)Pb_2I_7$ (**b**), $(PMA)_2(MA)_2Pb_3I_{10}$ (**e**), and $(HA)_2(GA)Pb_2I_7$ (**h**) NWs. The emission spectra of $(PMA)_2(MA)Pb_2I_7$ (**c**), $(PMA)_2(MA)_2Pb_3I_{10}$ (**f**), and $(HA)_2(GA)Pb_2I_7$ (**i**) NWs under pump fluences around the lasing threshold. PMA^+ = phenylmethylammonium, HA^+ = hexylammmonium, MA^+ = methylammonium, and GA^+ = guanidinium.

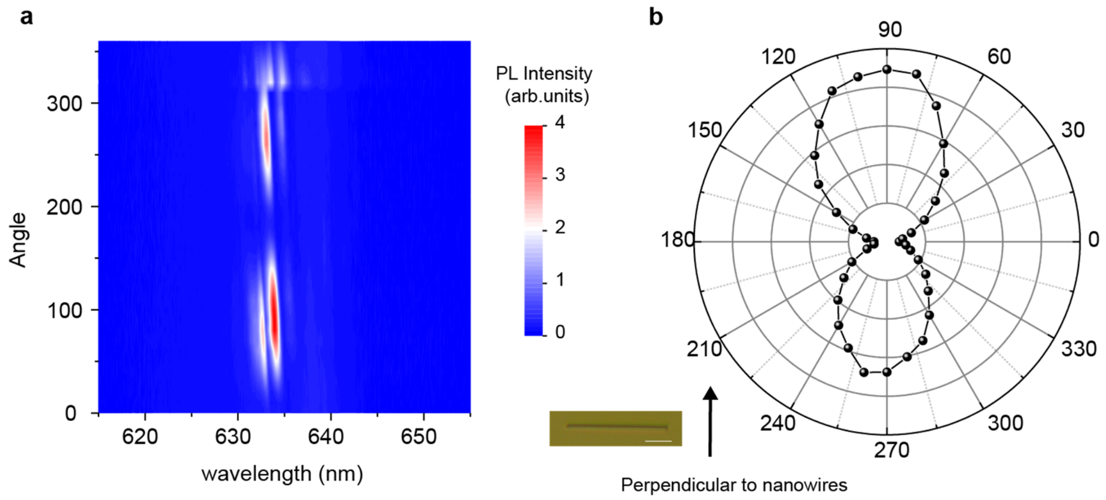

Supplementary Fig. 32. Lasing polarization of $(PMA)_2(MA)_2Pb_3I_{10}$ nanowires. **a**, color plot of the polarization resolved lasing spectra of $(PMA)_2(MA)_2Pb_3I_{10}$ NWs. **b**, polar plots of the PL intensity with different emission polarizations for $(PMA)_2(MA)_2Pb_3I_{10}$ NWs, showing the emission polarization perpendicular to the NW direction. Inset shows the alignment of nanowire, the scale bar is 5 μm. PMA^+ = phenylmethylammonium, and MA^+ = methylammonium.

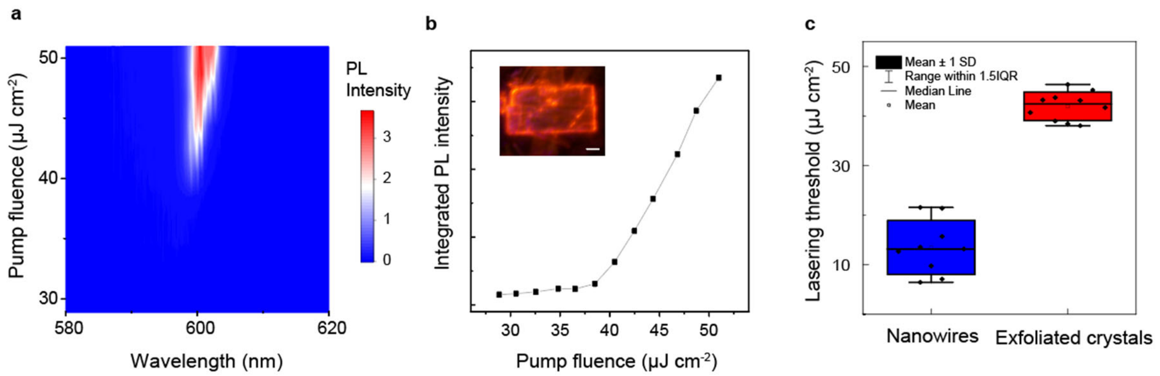

Supplementary Fig. 33. Lasing characterization of $(PMA)_2(MA)Pb_2I_7$ exfoliated crystals. **a**, 2D color plot of the emission spectra of a $(PMA)_2(MA)Pb_2I_7$ exfoliated crystal under different pump fluences. **b**, Integrated PL emission intensity versus the pump fluences, showing a lasing threshold ~39 μJ cm^{-2}. Inset shows the optical image of the exfoliated crystal. Scale

bar is 2 μm. **c**, Comparison of the lasing threshold of NWs (9 samples) and exfoliated samples (10 samples). SD represents the standard deviation, and IQR represents the interquartile range. PMA^+ = phenylmethylammonium, and MA^+ = methylammonium.

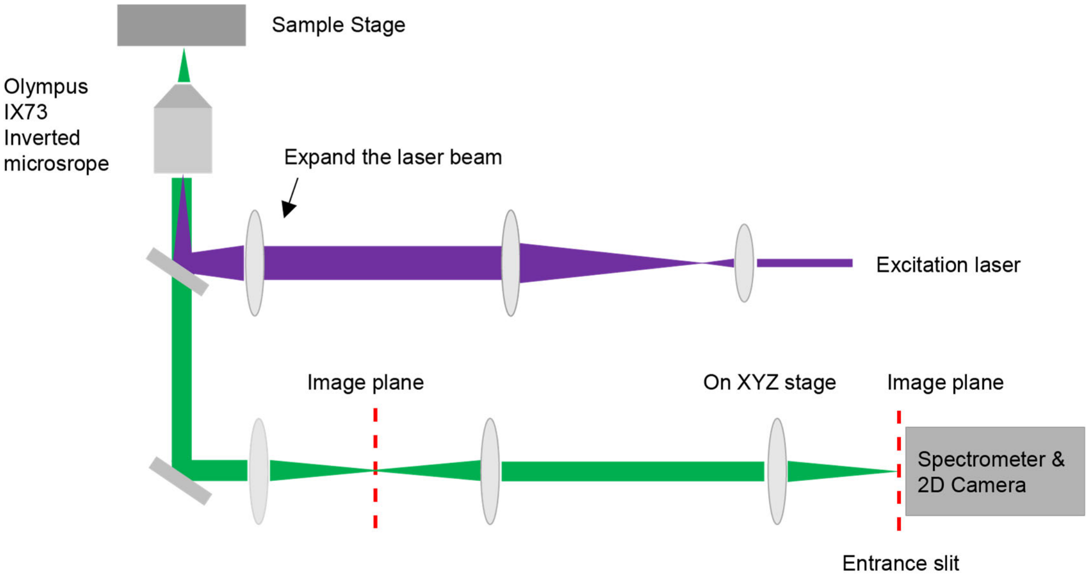

Supplementary Fig. 34. Optical setup for spatially resolved photoluminescence spectroscopy.

Supplementary Table 1. Comparisons of the structural phases and the associated intermolecular interactions in $(BA)_2PbI_4$, $(PA)_2PbI_4$, and $(HA)_2PbI_4$.

		$(BA)_2PbI_4$		$(PA)_2PbI_4$		$(HA)_2PbI_4$	
Phase transition temperature		274 K		319 K		268 K	
Phase		Low-T phase (223 K)	High-T phase (293 K)	Low-T phase (293 K)	High-T phase (333 K)	Low-T phase (173 K)	High-T phase (293 K)
Space group		Pbca	Pbca	$P2_1/a$	Pbca	$P2_1/a$	Pbca
In-plane *a* (Å)		8.428	8.876	8.672	9.008	8.643	8.941
In-plane *b* (Å)		8.986	8.692	8.930	8.731	8.845	8.687
C1	Distances along shorter / longer axis (Å)	5.37 / 7.09	5.83 / 6.61	6.63 / 6.09	5.93 / 6.63	5.74 / 6.66	5.86 / 6.63
	Anisotropic ratio*	1.32	1.13	0.91	1.12	1.16	1.13
C2	Distances along shorter / longer axis (Å)	5.69 / 6.68	5.99 / 6.44	4.79 / 8.14	6.09 / 6.46	6.06 / 6.31	6.04 / 6.43
	Anisotropic ratio	1.17	1.08	1.70	1.06	1.04	1.06
C3	Distances along shorter / longer axis (Å)	4.78 / 8.16	4.81 / 8.08	4.72 / 8.28	4.70 / 8.47	4.85 / 7.93	4.95 / 7.87
	Anisotropic ratio	1.71	1.68	1.75	1.80	1.64	1.59
C4	Distances along shorter / longer axis (Å)	5.07 / 7.56	4.97 / 7.79	4.40 / 10.62	4.94 / 8.00	5.26 / 7.31	5.27 / 7.37
	Anisotropic ratio	1.49	1.57	2.41	1.62	1.40	1.40
C5	Distances along shorter / longer axis (Å)	-	-	4.38 / 10.49	5.41 / 7.27	4.43 / 8.98	4.58 / 8.65
	Anisotropic ratio	-	-	2.39	1.34	2.03	1.89
C6	Distances along shorter / longer axis (Å)	-	-	-	-	4.65 / 8.34	4.85 / 8.05

	Anisotropic ratio	-	-	-	-	1.79	1.66

Note that the shaded columns are the room-temperature phases. The carbon atom adjacent to the ammonium group is designated as C1, while the carbon atom at the terminus of the alkyl chain is labelled as C4(BA)/C5(PA)/C6(HA). * The anisotropic ratio is defined as the ratio of the carbon-carbon distance between neighboring alkylammonium cations along the longer axis to the corresponding distance along the shorter axis.

Supplementary Table 2. Comparison of the carbon-carbon distances along the shorter-axis and longer-axis between two neighboring cations in $(HA)_2(MA)\ Pb_2I_7$ and $(HA)_2(GA)\ Pb_2I_7$.

Perovskite	Carbon atom	Distance along the shorter axis/Å	Distance along the longer axis/Å	Anisotropic ratio (longer/shorter)	In-plane a-axis/Å	In-plane b-axis/Å
$(HA)_2(MA)$ Pb_2I_7	C1	5.63	6.80	1.21	8.84	8.70
	C2	6.06	6.63	1.10		
	C3	4.85	7.96	1.64		
	C4	5.22	7.35	1.41		
	C5	4.44	9.03	2.04		
	C6	4.64	8.40	1.81		
$(HA)_2(GA)$ Pb_2I_7	C1	5.58/5.62	7.05/7.04	1.26/1.25	9.03	8.82
	C2	5.99/5.99	6.74/6.64	1.13/1.11		
	C3	4.48/4.66	8.66/8.66	1.93/1.86		
	C4	4.81/5.08	8.58/8.37	1.78/1.65		
	C5	4.07/4.46	10.67/10.63	2.62/2.38		
	C6	4.48/4.99	10.58/10.35	2.36/2.07		

* The HA^+ cations are disordered in the $(HA)_2(GA)Pb_2I_7$, and thus there are two set of data. The carbon

atom adjacent to the ammonium group is designated as C1, while the carbon atom at the terminus of the alkyl chain is labelled as C6.

Supplementary Table 3. Recipes for all 2D perovskite precursor solutions.

Phases	BX_2	LA	A	HX	H_3PO_2
$(PMA)_2PbI_4$	1.0 mmol PbI_2	1.0 mmol	/	5 mL	1 mL
$(PEA)_2PbI_4$	1.0 mmol PbI_2	1.0 mmol	/	9 mL	1 mL
$(BA)_2PbI_4$	1.0 mmol PbI_2	2.0 mmol	/	5 mL	1 mL
$(ABA)_2PbI_4$	1.0 mmol PbI_2	2.0 mmol	/	2 mL	0.5 mL
$(PA)_2PbI_4$	1.0 mmol PbI_2	2.0 mmol	/	5 mL	1 mL
$(HA)_2PbI_4$	1.0 mmol PbI_2	2.0 mmol	/	5 mL	1 mL
$(MBA)_2PbI_4$	1.0 mmol PbI_2	1.0 mmol	/	5 mL	1 mL
$(2FPMA)_2PbI_4$	1.0 mmol PbI_2	1.0 mmol	/	5 mL	1 mL
$(4FPMA)_2PbI_4$	1.0 mmol PbI_2	1.0 mmol	/	5 mL	1 mL
$(4ClPMA)_2PbI_4$	1.0 mmol PbI_2	1.0 mmol	/	5 mL	1 mL
$(4CF_3PMA)_2PbI_4$	1.0 mmol PbI_2	1.0 mmol	/	5 mL	1 mL
$(2CF_3PEA)_2PbI_4$	0.25 mmol PbI_2	0.5 mmol	/	9 mL	1 mL
$(2BrPEA)_2PbI_4$	0.25 mmol PbI_2	0.5 mmol	/	6 mL	1 mL
$(DFP)_2PbI_4$	1.0 mmol PbI_2	1.2 mmol	/	6 mL	1 mL
$(DFPD)_2PbI_4$	1.0 mmol PbI_2	1.2 mmol	/	6 mL	1 mL
$(PMA)_2SnI_4$	1.0 mmol $Sn(Ac)_2$	1.0 mmol	/	5 mL	1.5 mL
$(PA)_2SnI_4$	1.0 mmol $Sn(Ac)_2$	1.0 mmol	/	5 mL	1.5 mL
$(MBA)_2SnI_4$	1.0 mmol $Sn(Ac)_2$	2.0 mmol	/	3 mL	1.5 mL
$(2FPMA)_2SnI_4$	1.0 mmol $Sn(Ac)_2$	1.0 mmol	/	3 mL	1.5 mL
$(DFP)_2SnI_4$	1.0 mmol $Sn(Ac)_2$	0.9 mmol	/	5 mL	1.5 mL
$(DFPD)_2SnI_4$	1.0 mmol $Sn(Ac)_2$	0.9 mmol	/	3 mL	1 mL
$(PMA)_2PbBr_4$	1.0 mmol $PbBr_2$	2.0 mmol	/	5 mL HBr	1 mL
$(2BrPEA)_2PbBr_4$	0.5 mmol $PbBr_2$	1.0 mmol	/	10 mL HBr	1 mL
$(2CF_3PEA)_2CuCl_4$	0.5 mmol $CuCl_2$	1.0 mmol	/	0.2 mL HCl +3mL H_2O	/
$(CH_2O_2PEA)_2CdCl_4$	0.5 mmol $CdCl_2$	1.0 mmol	/	0.2 mL HCl +3mL H_2O	/
$(PMA)_2(MA)Pb_2I_7$	1.5 mmol PbI_2	1.0 mmol	8.0 mmol MA·HCl	5 mL	1 mL
$(PMA)_2(MA)_2Pb_3I_{10}$	2.5 mmol PbI_2	1.0 mmol	9.0 mmol MA·HCl	5 mL	1 mL

$(PMA)_2(FA)Pb_2I_7$	1.0 mmol PbI_2	1.0 mmol	1.3 mmol FA·HCl	5 mL	1 mL
$(PA)_2(MA)Pb_2I_7$	1.0 mmol PbI_2	0.6 mmol	0.6 mmol MA·HCl	3 mL	0.5 mL
$(PA)_2(FA)Pb_2I_7$	1.0 mmol PbI_2	1.0 mmol	0.6 mmol FA·HCl	4.5 mL	0.5 mL
$(PA)_2(ATA)Pb_2I_7$	1.0 mmol PbI_2	1.2 mmol	1.0 mmol ATA·HCl	6 mL	0.5 mL
$(HA)_2(MA)Pb_2I_7$	1.0 mmol PbI_2	0.9 mmol	1.0 mmol MA·HCl	2.5 mL	0.3 mL
$(HA)_2(GA)Pb_2I_7$	1.0 mmol PbI_2	0.5 mmol	1.0 mmol GA·HCl	3 mL	0.3 mL
