# Supplementary material for: Two-dimensional organic-inorganic hybrid perovskite quantum-well nanowires enabled by directional noncovalent intermolecular interactions
Source: Nat Commun. 2025 Mar 27;16:2997. doi: 10.1038/s41467-025-58166-x (PMC11950231; doi:10.1038/s41467-025-58166-x)
Supplement: Supplementary file 2 — Description of Additional Supplementary Files [file 41467_2025_58166_MOESM2_ESM.pdf]

Description of Additional Supplementary Files

Supplementary Movie 1, Real-time video of the growth of $(PMA)_2PbI_4$ nanowires, PMA^+ = phenylmethylammonium. The size of the window is 380 μm × 280 μm.

Supplementary Data 1, Cif files and checkcif files of the representative 2D perovskite crystals.
