# Supplementary material for: Two-dimensional organic-inorganic hybrid perovskite quantum-well nanowires enabled by directional noncovalent intermolecular interactions
Source: Nat Commun. 2025 Mar 27;16:2997. doi: 10.1038/s41467-025-58166-x (PMC11950231; doi:10.1038/s41467-025-58166-x)

checkCIF/PLATON report

Structure factors have been supplied for datablock(s) 2CF3PEA2CuCl4

THIS REPORT IS FOR GUIDANCE ONLY. IF USED AS PART OF A REVIEW PROCEDURE FOR PUBLICATION, IT SHOULD NOT REPLACE THE EXPERTISE OF AN EXPERIENCED CRYSTALLOGRAPHIC REFEREE.

No syntax errors found. CIF dictionary Interpreting this report

Datablock: 2CF3PEA2CuCl4

Bond precision: C-C = 0.0042 A Wavelength=0.71073

Cell:	a=18.1270(7)	b=8.2533(5)	c=7.6658(3)
	alpha=90	beta=90.631(4)	gamma=90
Temperature:	290 K		

	Calculated	Reported
Volume	1146.79(9)	1146.79(9)
Space group	P 21/c	P 1 21/c 1
Hall group	-P 2ybc	-P 2ybc
Moiety formula	2(C9 H11 F3 N), Cl4 Cu	Cl4 Cu, 2(C9 H11 F3 N)
Sum formula	C18 H22 Cl4 Cu F6 N2	C18 H22 Cl4 Cu F6 N2
Mr	585.73	585.71
Dx,g cm-3	1.696	1.696
Z	2	2
Mu (mm-1)	1.476	1.476
F000	590.0	590.0
F000’	592.10	
h,k,lmax	25,11,10	25,11,10
Nref	3198	2893
Tmin,Tmax	0.893,0.934	0.316,1.000
Tmin’	0.450	

Correction method= # Reported T Limits: Tmin=0.316 Tmax=1.000
AbsCorr = GAUSSIAN

Data completeness= 0.905 Theta(max)= 29.474

R(reflections)= 0.0501(2080) wR2(reflections)= 0.1439(2893)

S = 1.003 Npar= 143

The following ALERTS were generated. Each ALERT has the format
test-name_ALERT_alert-type_alert-level.
Click on the hyperlinks for more details of the test.

Alert level C

```
PLAT042_ALERT_1_C Calc. and Reported MoietyFormula Strings  Differ     Please Check
              Calc: 2(C9 H11 F3 N), Cl4 Cu
              Rep.: Cl4 Cu, 2(C9 H11 F3 N)
PLAT094_ALERT_2_C Ratio of Maximum / Minimum Residual Density ....       2.17 Report
PLAT906_ALERT_3_C Large K Value in the Analysis of Variance ......      3.294 Check
```

Alert level G

```
PLAT007_ALERT_5_G Number of Unrefined Donor-H Atoms .............          3 Report
              H1A   H1B   H1C
PLAT012_ALERT_1_G N.O.K.   _shelx_res_checksum Found in CIF ......     Please Check
PLAT242_ALERT_2_G Low    'MainMol' Ueq as Compared to Neighbors of       C00D Check
PLAT720_ALERT_4_G Number of Unusual/Non-Standard Labels ..........         19 Note
              Cl02    Cl03    C007    C008    C00A    H00A    H00B    C00B
              H00C    C00C    H00D    H00E    C00D    C00E    H00F    C00F
              H00G    C00G    H00H
PLAT910_ALERT_3_G Missing # of FCF Reflection(s) Below Theta(Min).          1 Note
                1  0  0,
PLAT912_ALERT_4_G Missing # of FCF Reflections Above STh/L=  0.600        304 Note
PLAT941_ALERT_3_G Average HKL Measurement Multiplicity ..........         2.7 Low
PLAT969_ALERT_5_G The 'Henn et al.' R-Factor-gap value ..........       2.113 Note
              Predicted wR2: Based on SigI**2  6.81 or SHELX Weight 14.35
PLAT978_ALERT_2_G Number C-C Bonds with Positive Residual Density.          4 Info
```

0 **ALERT level A** = Most likely a serious problem - resolve or explain
0 **ALERT level B** = A potentially serious problem, consider carefully
3 **ALERT level C** = Check. Ensure it is not caused by an omission or oversight
9 **ALERT level G** = General information/check it is not something unexpected

2 ALERT type 1 CIF construction/syntax error, inconsistent or missing data
3 ALERT type 2 Indicator that the structure model may be wrong or deficient
3 ALERT type 3 Indicator that the structure quality may be low
2 ALERT type 4 Improvement, methodology, query or suggestion
2 ALERT type 5 Informative message, check

It is advisable to attempt to resolve as many as possible of the alerts in all categories. Often the minor alerts point to easily fixed oversights, errors and omissions in your CIF or refinement strategy, so attention to these fine details can be worthwhile. In order to resolve some of the more serious problems it may be necessary to carry out additional measurements or structure refinements. However, the purpose of your study may justify the reported deviations and the more serious of these should normally be commented upon in the discussion or experimental section of a paper or in the "special_details" fields of the CIF. checkCIF was carefully designed to identify outliers and unusual parameters, but every test has its limitations and alerts that are not important in a particular case may appear. Conversely, the absence of alerts does not guarantee there are no aspects of the results needing attention. It is up to the individual to critically assess their own results and, if necessary, seek expert advice.

Publication of your CIF in IUCr journals

A basic structural check has been run on your CIF. These basic checks will be run on all CIFs submitted for publication in IUCr journals (*Acta Crystallographica*, *Journal of Applied Crystallography*, *Journal of Synchrotron Radiation*); however, if you intend to submit to *Acta Crystallographica Section C* or *E* or *IUCrData*, you should make sure that full publication checks are run on the final version of your CIF prior to submission.

Publication of your CIF in other journals

Please refer to the *Notes for Authors* of the relevant journal for any special instructions relating to CIF submission.

PLATON version of 13/05/2024; check.def file version of 04/05/2024

Datablock 2CF3PEA2CuCl4 - ellipsoid plot

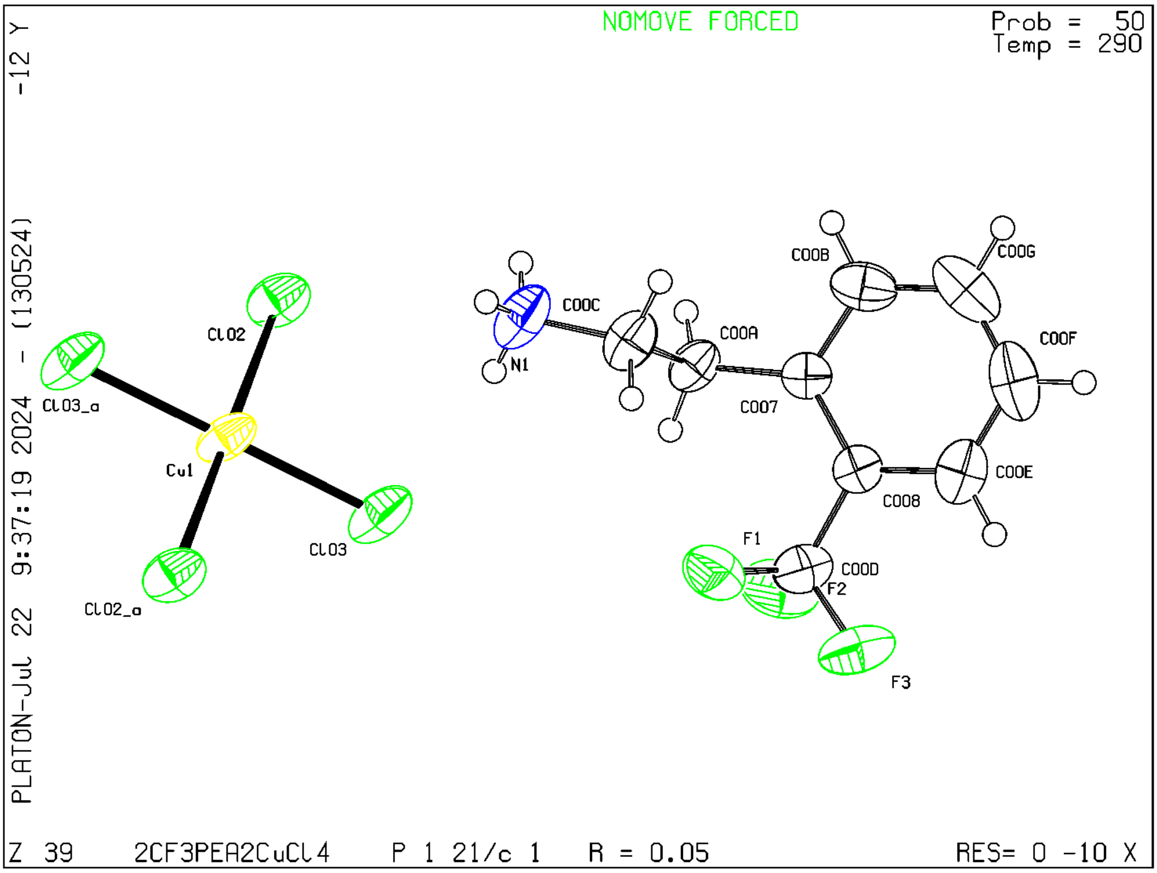

Supplement: Supplementary file 4 — Supplementary Data 1 [file 41467_2025_58166_MOESM4_ESM.zip › crystal structure cif and checkcif/(2CF3PEA)2CuCl4.pdf]
