# Supplementary material for: Two-dimensional organic-inorganic hybrid perovskite quantum-well nanowires enabled by directional noncovalent intermolecular interactions
Source: Nat Commun. 2025 Mar 27;16:2997. doi: 10.1038/s41467-025-58166-x (PMC11950231; doi:10.1038/s41467-025-58166-x)

checkCIF/PLATON report

Structure factors have been supplied for datablock(s) exp_14792

THIS REPORT IS FOR GUIDANCE ONLY. IF USED AS PART OF A REVIEW PROCEDURE FOR PUBLICATION, IT SHOULD NOT REPLACE THE EXPERTISE OF AN EXPERIENCED CRYSTALLOGRAPHIC REFEREE.

No syntax errors found.   CIF dictionary   Interpreting this report

Datablock: exp_14792

Bond precision:	C-C = 0.0145 A		Wavelength=0.71073
Cell:	a=23.2221(10)	b=9.3110(3)	c=8.9496(3)
	alpha=90	beta=90	gamma=90
Temperature:	290 K		

	Calculated	Reported
Volume	1935.09(12)	1935.10(13)
Space group	P n a 21	P n a 21
Hall group	P 2c -2n	P 2c -2n
Moiety formula	I4 Sn, 2(C4 H8 F2 N)	I4 Sn, 2(C4 H8 F2 N)
Sum formula	C8 H16 F4 I4 N2 Sn	C8 H16 F4 I4 N2 Sn
Mr	842.54	842.52
Dx,g cm-3	2.892	2.892
Z	4	4
Mu (mm-1)	7.726	7.726
F000	1504.0	1504.0
F000’	1494.55	
h,k,lmax	33,13,12	30,12,12
Nref	5877[3111]	3763
Tmin,Tmax	0.315,0.644	0.439,1.000
Tmin’	0.131	

Correction method= # Reported T Limits: Tmin=0.439 Tmax=1.000
AbsCorr = MULTI-SCAN

Data completeness= 1.21/0.64   Theta(max)= 30.414

R(reflections)= 0.0305(3293)   wR2(reflections)= 0.0763(3763)

S = 1.051   Npar= 172

The following ALERTS were generated. Each ALERT has the format
test-name_ALERT_alert-type_alert-level.
Click on the hyperlinks for more details of the test.

Alert level C

```
PLAT230_ALERT_2_C Hirshfeld Test Diff for    N00J     --C00B     .        6.8 s.u.
PLAT230_ALERT_2_C Hirshfeld Test Diff for    N00J     --C00I     .        6.3 s.u.
PLAT241_ALERT_2_C High   'MainMol' Ueq as Compared to Neighbors of       N00J Check
PLAT242_ALERT_2_C Low    'MainMol' Ueq as Compared to Neighbors of       C00G Check
PLAT242_ALERT_2_C Low    'MainMol' Ueq as Compared to Neighbors of       C00B Check
PLAT242_ALERT_2_C Low    'MainMol' Ueq as Compared to Neighbors of       C00C Check
PLAT342_ALERT_3_C Low Bond Precision on  C-C Bonds ...............     0.0145 Ang.
PLAT360_ALERT_2_C Short  C(sp3)-C(sp3) Bond  C00D     - C00E     .       1.43 Ang.
PLAT420_ALERT_2_C D-H Bond Without Acceptor  N00J     --H00O     .     Please Check
PLAT420_ALERT_2_C D-H Bond Without Acceptor  N00J     --H00P     .     Please Check
PLAT977_ALERT_2_C Check Negative Difference Density on H00P      .      -0.41 eA-3
```

Alert level G

```
PLAT003_ALERT_2_G Number of Uiso or U(i,j) Restrained non-H Atoms           5 Report
PLAT004_ALERT_5_G Polymeric Structure Found with Maximum Dimension          2 Info
PLAT005_ALERT_5_G No Embedded Refinement Details Found  in the CIF     Please Do !
PLAT007_ALERT_5_G Number of Unrefined Donor-H Atoms ..............          4 Report
              H00A  H00B  H00O  H00P
PLAT232_ALERT_2_G Hirshfeld Test Diff (M-X)  I003     --Sn00     .        5.8 s.u.
PLAT432_ALERT_2_G Short Inter X...Y Contact  I002     ..C00B     .       3.38 Ang.
                                                    x,y,z  =      1_555 Check
PLAT720_ALERT_4_G Number of Unusual/Non-Standard Labels ..........         35 Note
              Sn00    I002    I003    I004    I005    F006    F007    F008
              F009    N00A    H00A    H00B    C00B    H00I    H00J    C00C
              C00D    H00C    H00D    C00E    H00E    H00F    C00F    H00G
              H00H    C00G    C00H    H00K    H00L    C00I    H00M    H00N
              N00J    H00O    H00P
PLAT794_ALERT_5_G Tentative Bond Valency for Sn00      (II)      .       2.35 Info
PLAT860_ALERT_3_G Number of Least-Squares Restraints .............         32 Note
PLAT910_ALERT_3_G Missing # of FCF Reflection(s) Below Theta(Min).          1 Note
                2  0  0,
PLAT912_ALERT_4_G Missing # of FCF Reflections Above STh/L=  0.600        457 Note
PLAT915_ALERT_3_G No Flack x Check Done: Low Friedel Pair Coverage         42 %
PLAT950_ALERT_5_G Calculated (ThMax) and CIF-Reported Hmax Differ           3 Units
PLAT956_ALERT_1_G Calculated (ThMax) and Actual (FCF) Hmax Differ           3 Units
PLAT969_ALERT_5_G The 'Henn et al.' R-Factor-gap value ...........      1.461 Note
              Predicted wR2: Based on SigI**2  5.22 or SHELX Weight  7.26
PLAT978_ALERT_2_G Number C-C Bonds with Positive Residual Density.          0 Info
```

0 **ALERT level A** = Most likely a serious problem - resolve or explain
0 **ALERT level B** = A potentially serious problem, consider carefully
11 **ALERT level C** = Check. Ensure it is not caused by an omission or oversight
16 **ALERT level G** = General information/check it is not something unexpected

1 ALERT type 1 CIF construction/syntax error, inconsistent or missing data

```
14 ALERT type 2 Indicator that the structure model may be wrong or deficient
 4 ALERT type 3 Indicator that the structure quality may be low
 2 ALERT type 4 Improvement, methodology, query or suggestion
 6 ALERT type 5 Informative message, check
```

It is advisable to attempt to resolve as many as possible of the alerts in all categories. Often the minor alerts point to easily fixed oversights, errors and omissions in your CIF or refinement strategy, so attention to these fine details can be worthwhile. In order to resolve some of the more serious problems it may be necessary to carry out additional measurements or structure refinements. However, the purpose of your study may justify the reported deviations and the more serious of these should normally be commented upon in the discussion or experimental section of a paper or in the "special_details" fields of the CIF. checkCIF was carefully designed to identify outliers and unusual parameters, but every test has its limitations and alerts that are not important in a particular case may appear. Conversely, the absence of alerts does not guarantee there are no aspects of the results needing attention. It is up to the individual to critically assess their own results and, if necessary, seek expert advice.

Publication of your CIF in IUCr journals

A basic structural check has been run on your CIF. These basic checks will be run on all CIFs submitted for publication in IUCr journals (*Acta Crystallographica*, *Journal of Applied Crystallography*, *Journal of Synchrotron Radiation*); however, if you intend to submit to *Acta Crystallographica Section C* or *E* or *IUCrData*, you should make sure that full publication checks are run on the final version of your CIF prior to submission.

Publication of your CIF in other journals

Please refer to the *Notes for Authors* of the relevant journal for any special instructions relating to CIF submission.

PLATON version of 13/05/2024; check.def file version of 04/05/2024

Datablock exp_14792 - ellipsoid plot

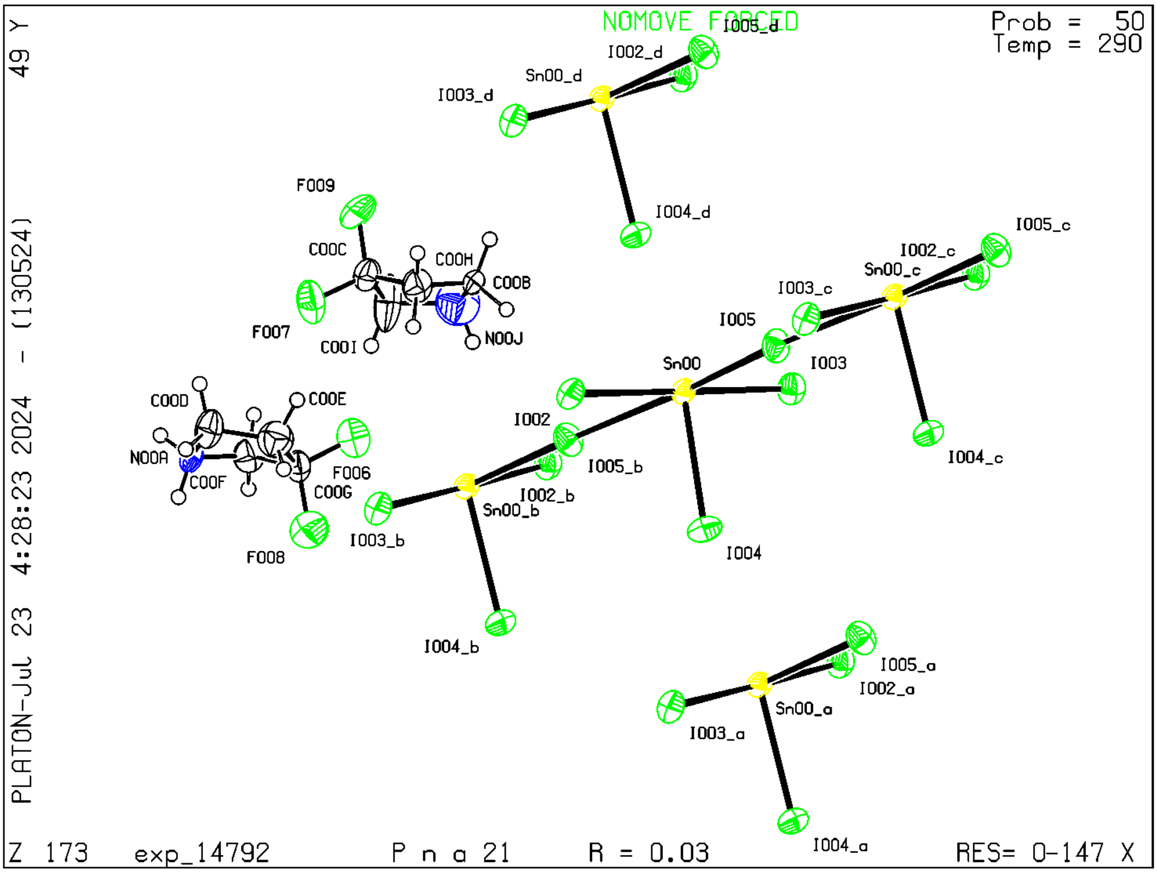

Supplement: Supplementary file 4 — Supplementary Data 1 [file 41467_2025_58166_MOESM4_ESM.zip › crystal structure cif and checkcif/(DFP)2SnI4 checkcif.pdf]
