# Supplementary material for: Two-dimensional organic-inorganic hybrid perovskite quantum-well nanowires enabled by directional noncovalent intermolecular interactions
Source: Nat Commun. 2025 Mar 27;16:2997. doi: 10.1038/s41467-025-58166-x (PMC11950231; doi:10.1038/s41467-025-58166-x)

checkCIF/PLATON report

Structure factors have been supplied for datablock(s) exp_14794

THIS REPORT IS FOR GUIDANCE ONLY. IF USED AS PART OF A REVIEW PROCEDURE FOR PUBLICATION, IT SHOULD NOT REPLACE THE EXPERTISE OF AN EXPERIENCED CRYSTALLOGRAPHIC REFEREE.

No syntax errors found. CIF dictionary Interpreting this report

Datablock: exp_14794

```
Bond precision:   C-C = 0.0150 A          Wavelength=0.71073

Cell:              a=25.2864(10)      b=8.8718(3)      c=9.5015(3)
                   alpha=90           beta=90          gamma=90
Temperature:       290 K

                 Calculated                   Reported
Volume           2131.53(13)                  2131.53(14)
Space group      P n a 21                     P n a 21
Hall group       P 2c -2n                     P 2c -2n
Moiety formula   I4 Sn, 2(C5 H10 F2 N)        I4 Sn, 2(C5 H10 F2 N)
Sum formula      C10 H20 F4 I4 N2 Sn          C10 H20 F4 I4 N2 Sn
Mr               870.59                       870.57
Dx,g cm-3        2.713                        2.713
Z                4                            4
Mu (mm-1)        7.019                        7.019
F000             1568.0                       1568.0
F000'            1558.55
h,k,lmax         37,13,14                     34,13,12
Nref             7128[ 3756]                  4584
Tmin,Tmax                                     0.833,1.000
Tmin'

Correction method= # Reported T Limits: Tmin=0.833 Tmax=1.000
AbsCorr = MULTI-SCAN

Data completeness= 1.22/0.64       Theta(max)= 31.584

R(reflections)= 0.0322( 4009)                          wR2(reflections)=
                                                       0.0857( 4584)
S = 1.062                  Npar= 211
```

The following ALERTS were generated. Each ALERT has the format
test-name_ALERT_alert-type_alert-level.
Click on the hyperlinks for more details of the test.

Alert level C

```
PLAT053_ALERT_1_C Minimum Crystal Dimension Missing (or Error) ...     Please Check
PLAT054_ALERT_1_C Medium  Crystal Dimension Missing (or Error) ...     Please Check
PLAT055_ALERT_1_C Maximum Crystal Dimension Missing (or Error) ...     Please Check
PLAT230_ALERT_2_C Hirshfeld Test Diff for    F8       --C2       .        5.2 s.u.
PLAT230_ALERT_2_C Hirshfeld Test Diff for    F9       --C2       .        5.7 s.u.
PLAT234_ALERT_4_C Large Hirshfeld Difference N22      --C18      .       0.16 Ang.
PLAT241_ALERT_2_C High   'MainMol' Ueq as Compared to Neighbors of        C30 Check
PLAT242_ALERT_2_C Low    'MainMol' Ueq as Compared to Neighbors of         Sn Check
PLAT242_ALERT_2_C Low    'MainMol' Ueq as Compared to Neighbors of         C2 Check
PLAT342_ALERT_3_C Low Bond Precision on  C-C Bonds ...............      0.015 Ang.
PLAT360_ALERT_2_C Short  C(sp3)-C(sp3) Bond  C13      - C18      .       1.42 Ang.
PLAT360_ALERT_2_C Short  C(sp3)-C(sp3) Bond  C16      - C30      .       1.43 Ang.
PLAT360_ALERT_2_C Short  C(sp3)-C(sp3) Bond  C3       - C29      .       1.42 Ang.
PLAT420_ALERT_2_C D-H Bond Without Acceptor  N21      --H21A     .     Please Check
PLAT911_ALERT_3_C Missing FCF Refl Between Thmin & STh/L=    0.600          3 Report
               20  7  0,  22  6  0,   8  3 10,
```

Alert level G

```
PLAT002_ALERT_2_G Number of Distance or Angle Restraints on AtSite         16 Note
PLAT003_ALERT_2_G Number of Uiso or U(i,j) Restrained non-H Atoms          19 Report
PLAT004_ALERT_5_G Polymeric Structure Found with Maximum Dimension          2 Info
PLAT005_ALERT_5_G No Embedded Refinement Details Found  in the CIF     Please Do !
PLAT007_ALERT_5_G Number of Unrefined Donor-H Atoms .............           4 Report
              H22A  H22B  H21A  H21B
PLAT112_ALERT_2_G ADDSYM Detects New (Pseudo) Symm. Elem         B         89 %Fit
PLAT230_ALERT_2_G Hirshfeld Test Diff for    F10A     --C9       .        5.1 s.u.
PLAT232_ALERT_2_G Hirshfeld Test Diff (M-X)  I002     --Sn       .        9.0 s.u.
PLAT232_ALERT_2_G Hirshfeld Test Diff (M-X)  I004     --Sn       .        7.0 s.u.
PLAT242_ALERT_2_G Low    'MainMol' Ueq as Compared to Neighbors of         C9 Check
PLAT301_ALERT_3_G Main Residue  Disorder ..............(Resd    2)        25% Note
PLAT720_ALERT_4_G Number of Unusual/Non-Standard Labels ..........          4 Note
              I002    I003    I004    I005
PLAT764_ALERT_4_G Overcomplete CIF Bond List Detected (Rep/Expd) .       1.14 Ratio
PLAT779_ALERT_4_G Suspect or Irrelevant (Bond) Angle(s) in CIF ...      32.50 Deg.
              F12  -C9   -F12A    1_555   1_555   1_555 ......     #   24 Check
PLAT779_ALERT_4_G Suspect or Irrelevant (Bond) Angle(s) in CIF ...      26.40 Deg.
              F10A -C9   -F10     1_555   1_555   1_555 ......     #   29 Check
PLAT789_ALERT_4_G Atoms with Negative _atom_site_disorder_group  #          2 Check
PLAT794_ALERT_5_G Tentative Bond Valency for Sn        (II)      .       2.32 Info
PLAT860_ALERT_3_G Number of Least-Squares Restraints .............        315 Note
PLAT910_ALERT_3_G Missing # of FCF Reflection(s) Below Theta(Min).          1 Note
                2  0  0,
PLAT912_ALERT_4_G Missing # of FCF Reflections Above STh/L=  0.600        816 Note
PLAT915_ALERT_3_G No Flack x Check Done: Low Friedel Pair Coverage         51 %
PLAT950_ALERT_5_G Calculated (ThMax) and CIF-Reported Hmax Differ           3 Units
PLAT952_ALERT_5_G Calculated (ThMax) and CIF-Reported Lmax Differ.          2 Units
PLAT956_ALERT_1_G Calculated (ThMax) and Actual (FCF) Hmax Differ           3 Units
PLAT958_ALERT_1_G Calculated (ThMax) and Actual (FCF) Lmax Differ.          2 Units
PLAT969_ALERT_5_G The 'Henn et al.' R-Factor-gap value ..........       2.353 Note
```

```
             Predicted wR2: Based on SigI**2  3.64 or SHELX Weight  8.07
PLAT978_ALERT_2_G Number C-C Bonds with Positive Residual Density.         0 Info
```

```
  0 ALERT level A = Most likely a serious problem - resolve or explain
  0 ALERT level B = A potentially serious problem, consider carefully
 15 ALERT level C = Check. Ensure it is not caused by an omission or oversight
 27 ALERT level G = General information/check it is not something unexpected

  5 ALERT type 1 CIF construction/syntax error, inconsistent or missing data
 17 ALERT type 2 Indicator that the structure model may be wrong or deficient
  6 ALERT type 3 Indicator that the structure quality may be low
  7 ALERT type 4 Improvement, methodology, query or suggestion
  7 ALERT type 5 Informative message, check
```

It is advisable to attempt to resolve as many as possible of the alerts in all categories. Often the minor alerts point to easily fixed oversights, errors and omissions in your CIF or refinement strategy, so attention to these fine details can be worthwhile. In order to resolve some of the more serious problems it may be necessary to carry out additional measurements or structure refinements. However, the purpose of your study may justify the reported deviations and the more serious of these should normally be commented upon in the discussion or experimental section of a paper or in the "special_details" fields of the CIF. checkCIF was carefully designed to identify outliers and unusual parameters, but every test has its limitations and alerts that are not important in a particular case may appear. Conversely, the absence of alerts does not guarantee there are no aspects of the results needing attention. It is up to the individual to critically assess their own results and, if necessary, seek expert advice.

Publication of your CIF in IUCr journals

A basic structural check has been run on your CIF. These basic checks will be run on all CIFs submitted for publication in IUCr journals (*Acta Crystallographica*, *Journal of Applied Crystallography*, *Journal of Synchrotron Radiation*); however, if you intend to submit to *Acta Crystallographica Section C* or *E* or *IUCrData*, you should make sure that full publication checks are run on the final version of your CIF prior to submission.

Publication of your CIF in other journals

Please refer to the *Notes for Authors* of the relevant journal for any special instructions relating to CIF submission.

PLATON version of 13/05/2024; check.def file version of 04/05/2024

Datablock exp_14794 - ellipsoid plot

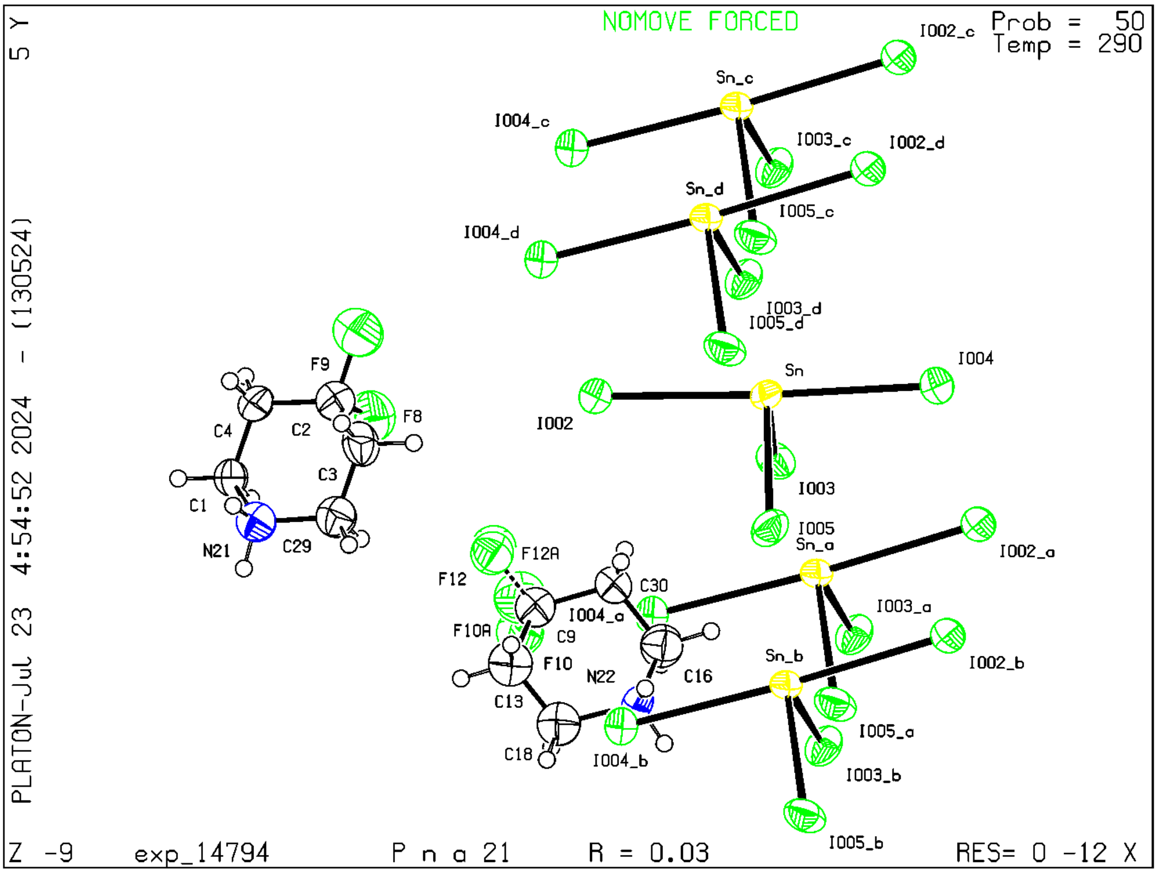

Supplement: Supplementary file 4 — Supplementary Data 1 [file 41467_2025_58166_MOESM4_ESM.zip › crystal structure cif and checkcif/(DFPD)2SnI4 checkcif.pdf]
