# Supplementary material for: Two-dimensional organic-inorganic hybrid perovskite quantum-well nanowires enabled by directional noncovalent intermolecular interactions
Source: Nat Commun. 2025 Mar 27;16:2997. doi: 10.1038/s41467-025-58166-x (PMC11950231; doi:10.1038/s41467-025-58166-x)

checkCIF/PLATON report

Structure factors have been supplied for datablock(s) exp_14789

THIS REPORT IS FOR GUIDANCE ONLY. IF USED AS PART OF A REVIEW PROCEDURE FOR PUBLICATION, IT SHOULD NOT REPLACE THE EXPERTISE OF AN EXPERIENCED CRYSTALLOGRAPHIC REFEREE.

No syntax errors found. CIF dictionary Interpreting this report

Datablock: exp_14789

Bond precision:	C-C = 0.0070 A		Wavelength=0.71073
Cell:	a=9.0940(3)	b=8.6663(3)	c=28.7329(11)
	alpha=90	beta=90	gamma=90
Temperature:	290 K		

	Calculated	Reported
Volume	2264.48(14)	2264.48(14)
Space group	P b c a	P b c a
Hall group	-P 2ac 2ab	-P 2ac 2ab
Moiety formula	I4 Sn, 2(C7 H10 N)	I4 Sn, 2(C7 H10 N)
Sum formula	C14 H20 I4 N2 Sn	C14 H20 I4 N2 Sn
Mr	842.63	842.61
Dx,g cm-3	2.472	2.472
Z	4	4
Mu (mm-1)	6.579	6.579
F000	1520.0	1520.0
F000'	1510.34	
h,k,lmax	12,11,39	12,11,38
Nref	3145	2893
Tmin,Tmax		0.617,1.000
Tmin'		

Correction method= # Reported T Limits: Tmin=0.617 Tmax=1.000
AbsCorr = MULTI-SCAN

Data completeness= 0.920 Theta(max)= 29.476

R(reflections)= 0.0268(2368) wR2(reflections)= 0.0549(2893)

S = 1.066 Npar= 98

```
The following ALERTS were generated. Each ALERT has the format
       test-name_ALERT_alert-type_alert-level.
Click on the hyperlinks for more details of the test.
```

Alert level C

```
PLAT053_ALERT_1_C Minimum Crystal Dimension Missing (or Error) ...     Please Check
PLAT054_ALERT_1_C Medium  Crystal Dimension Missing (or Error) ...     Please Check
PLAT055_ALERT_1_C Maximum Crystal Dimension Missing (or Error) ...     Please Check
PLAT241_ALERT_2_C High   'MainMol' Ueq as Compared to Neighbors of       C006 Check
PLAT242_ALERT_2_C Low    'MainMol' Ueq as Compared to Neighbors of       C005 Check
PLAT331_ALERT_2_C Small Aver Phenyl C-C Dist C005     --C008      .       1.36 Ang.
PLAT420_ALERT_2_C D-H Bond Without Acceptor  N004     --H00A      .    Please Check
PLAT906_ALERT_3_C Large K Value in the Analysis of Variance ......      2.398 Check
```

Alert level G

```
PLAT004_ALERT_5_G Polymeric Structure Found with Maximum Dimension          2 Info
PLAT005_ALERT_5_G No Embedded Refinement Details Found  in the CIF     Please Do !
PLAT007_ALERT_5_G Number of Unrefined Donor-H Atoms .............           3 Report
              H00A  H00B  H00C
PLAT232_ALERT_2_G Hirshfeld Test Diff (M-X)  I002     --Sn00      .       11.5 s.u.
PLAT232_ALERT_2_G Hirshfeld Test Diff (M-X)  I002     --Sn00_b    .       14.5 s.u.
PLAT232_ALERT_2_G Hirshfeld Test Diff (M-X)  I003     --Sn00      .       19.0 s.u.
PLAT720_ALERT_4_G Number of Unusual/Non-Standard Labels ..........          21 Note
              Sn00    I002    I003    N004    H00A    H00B    H00C    C005
              C006    H00D    H00E    C007    H007    C008    H008    C009
              H009    C00A    H00F    C00B    H00G
PLAT764_ALERT_4_G Overcomplete CIF Bond List Detected (Rep/Expd) .       1.27 Ratio
PLAT794_ALERT_5_G Tentative Bond Valency for Sn00      (II)      .       2.23 Info
PLAT910_ALERT_3_G Missing # of FCF Reflection(s) Below Theta(Min).          1 Note
               0  0  2,
PLAT912_ALERT_4_G Missing # of FCF Reflections Above STh/L=  0.600        246 Note
PLAT969_ALERT_5_G The 'Henn et al.' R-Factor-gap value ..........      1.898 Note
              Predicted wR2: Based on SigI**2  2.89 or SHELX Weight  5.15
PLAT978_ALERT_2_G Number C-C Bonds with Positive Residual Density.          0 Info
```

```
   0 ALERT level A = Most likely a serious problem - resolve or explain
   0 ALERT level B = A potentially serious problem, consider carefully
   8 ALERT level C = Check. Ensure it is not caused by an omission or oversight
  13 ALERT level G = General information/check it is not something unexpected

   3 ALERT type 1 CIF construction/syntax error, inconsistent or missing data
   8 ALERT type 2 Indicator that the structure model may be wrong or deficient
   2 ALERT type 3 Indicator that the structure quality may be low
   3 ALERT type 4 Improvement, methodology, query or suggestion
   5 ALERT type 5 Informative message, check
```

It is advisable to attempt to resolve as many as possible of the alerts in all categories. Often the minor alerts point to easily fixed oversights, errors and omissions in your CIF or refinement strategy, so attention to these fine details can be worthwhile. In order to resolve some of the more serious problems it may be necessary to carry out additional measurements or structure refinements. However, the purpose of your study may justify the reported deviations and the more serious of these should normally be commented upon in the discussion or experimental section of a paper or in the "special_details" fields of the CIF. checkCIF was carefully designed to identify outliers and unusual parameters, but every test has its limitations and alerts that are not important in a particular case may appear. Conversely, the absence of alerts does not guarantee there are no aspects of the results needing attention. It is up to the individual to critically assess their own results and, if necessary, seek expert advice.

Publication of your CIF in IUCr journals

A basic structural check has been run on your CIF. These basic checks will be run on all CIFs submitted for publication in IUCr journals (*Acta Crystallographica*, *Journal of Applied Crystallography*, *Journal of Synchrotron Radiation*); however, if you intend to submit to *Acta Crystallographica Section C* or *E* or *IUCrData*, you should make sure that full publication checks are run on the final version of your CIF prior to submission.

Publication of your CIF in other journals

Please refer to the *Notes for Authors* of the relevant journal for any special instructions relating to CIF submission.

PLATON version of 13/05/2024; check.def file version of 04/05/2024

Datablock exp_14789 - ellipsoid plot

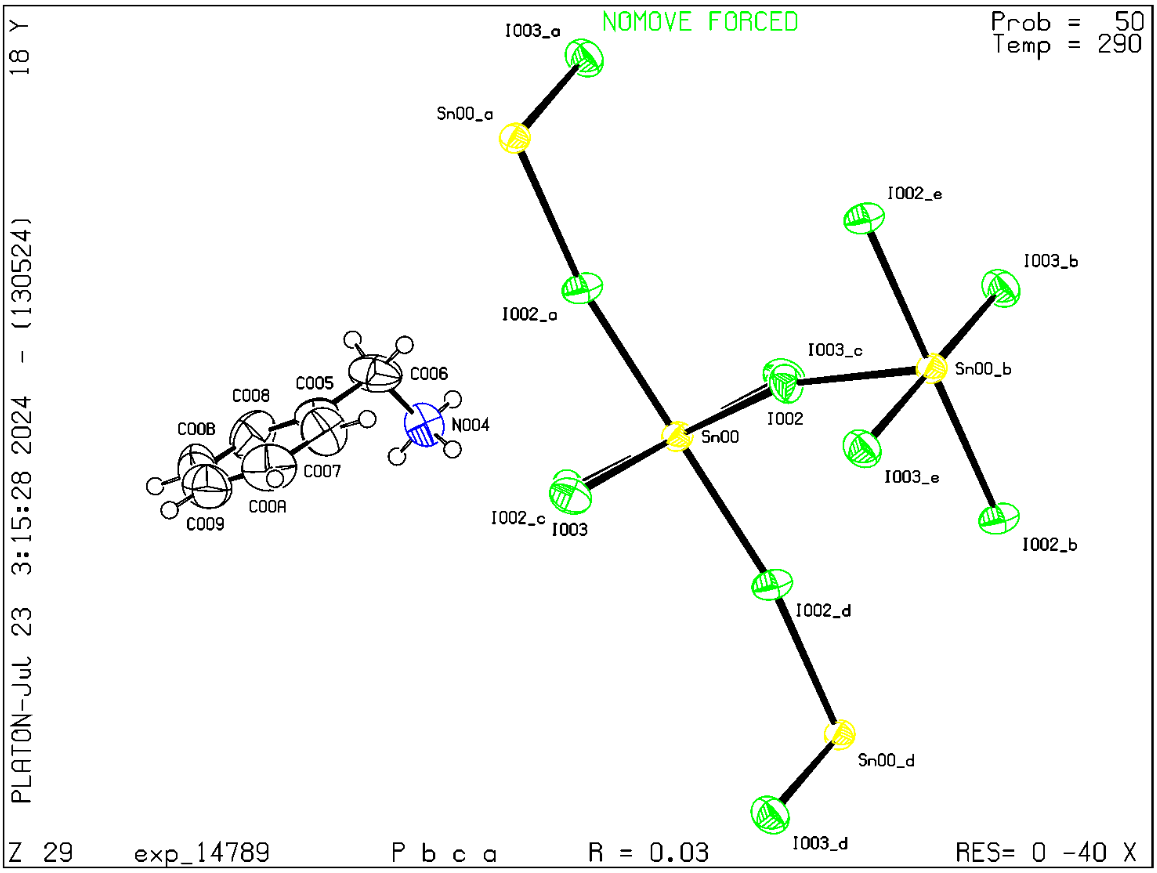

Supplement: Supplementary file 4 — Supplementary Data 1 [file 41467_2025_58166_MOESM4_ESM.zip › crystal structure cif and checkcif/(PMA)2SnI4 checkcif.pdf]
