# Supplementary material for: Two-dimensional organic-inorganic hybrid perovskite quantum-well nanowires enabled by directional noncovalent intermolecular interactions
Source: Nat Commun. 2025 Mar 27;16:2997. doi: 10.1038/s41467-025-58166-x (PMC11950231; doi:10.1038/s41467-025-58166-x)
Supplement: Supplementary file 5 — Reporting Summary [file 41467_2025_58166_MOESM5_ESM.pdf]

Corresponding author(s): Yongping Fu

Lasing Reporting Summary

Nature Research wishes to improve the reproducibility of the work that we publish. This form is intended for publication with all accepted papers reporting claims of lasing and provides structure for consistency and transparency in reporting. Some list items might not apply to an individual manuscript, but all fields must be completed for clarity.

For further information on Nature Research policies, including our data availability policy, see Authors & Referees.

• Experimental design

Please check: are the following details reported in the manuscript?

1. Threshold

Plots of device output power versus pump power over a wide range of values indicating a clear threshold

- [x] Yes
- [ ] No

Fig. 5e and Supplementary Figs 31 & 32

2. Linewidth narrowing

Plots of spectral power density for the emission at pump powers below, around, and above the lasing threshold, indicating a clear linewidth narrowing at threshold

- [x] Yes
- [ ] No

Fig. 5e and Supplementary Fig. 31

Resolution of the spectrometer used to make spectral measurements

- [x] Yes
- [ ] No

This information can be found in "Method"

3. Coherent emission

Measurements of the coherence and/or polarization of the emission

- [x] Yes
- [ ] No

Supplementary Fig 33

4. Beam spatial profile

Image and/or measurement of the spatial shape and profile of the emission, showing a well-defined beam above threshold

- [x] Yes
- [ ] No

Fig. 5f

5. Operating conditions

Description of the laser and pumping conditions
Continuous-wave, pulsed, temperature of operation

- [x] Yes
- [ ] No

This information can be found in "Method"

Threshold values provided as density values (e.g. W cm^{-2} or J cm^{-2}) taking into account the area of the device

- [x] Yes
- [ ] No

Fig. 5e and Supplementary Figs 31& 32

6. Alternative explanations

Reasoning as to why alternative explanations have been ruled out as responsible for the emission characteristics
e.g. amplified spontaneous, directional scattering; modification of fluorescence spectrum by the cavity

- [x] Yes
- [ ] No

Fig. 5e and Supplementary Figs 31, 32, and 33

7. Theoretical analysis

Theoretical analysis that ensures that the experimental values measured are realistic and reasonable
e.g. laser threshold, linewidth, cavity gain-loss, efficiency

- [ ] Yes
- [x] No

We have performed lasing experiments on exfoliated crystals as control, which show similar lasing performance as previous reports. So we do not need the theoretical analysis.

8. Statistics

Number of devices fabricated and tested

- [x] Yes
- [ ] No

Supplementary Fig 32

Statistical analysis of the device performance and lifetime (time to failure)

- [x] Yes
- [ ] No

Supplementary Fig 32
