# Supplementary material for: Two-dimensional organic-inorganic hybrid perovskite quantum-well nanowires enabled by directional noncovalent intermolecular interactions
Source: Nat Commun. 2025 Mar 27;16:2997. doi: 10.1038/s41467-025-58166-x (PMC11950231; doi:10.1038/s41467-025-58166-x)

checkCIF/PLATON report

Structure factors have been supplied for datablock(s) exp_14795

THIS REPORT IS FOR GUIDANCE ONLY. IF USED AS PART OF A REVIEW PROCEDURE FOR PUBLICATION, IT SHOULD NOT REPLACE THE EXPERTISE OF AN EXPERIENCED CRYSTALLOGRAPHIC REFEREE.

No syntax errors found. CIF dictionary Interpreting this report

Datablock: exp_14795

```
Bond precision:   C-C = 0.0224 A           Wavelength=0.71073

Cell:              a=9.1042(6)      b=8.6662(3)      c=29.0327(16)
                   alpha=90         beta=90          gamma=90
Temperature:       290 K

                Calculated                   Reported
Volume          2290.7(2)                    2290.6(2)
Space group     P b c a                      P b c a
Hall group      -P 2ac 2ab                   -P 2ac 2ab
Moiety formula  I4 Sn, 2(C7 H9 F N)          I4 Sn, 2(C7 H9 F N)
Sum formula     C14 H18 F2 I4 N2 Sn          C14 H18 F2 I4 N2 Sn
Mr              878.61                       878.59
Dx,g cm-3       2.548                        2.548
Z               4                            4
Mu (mm-1)       6.521                        6.521
F000            1584.0                       1584.0
F000'           1574.45
h,k,lmax        12,12,40                     12,11,39
Nref            3178                         2902
Tmin,Tmax                                    0.561,1.000
Tmin'

Correction method= # Reported T Limits: Tmin=0.561 Tmax=1.000
AbsCorr = MULTI-SCAN

Data completeness= 0.913           Theta(max)= 29.481

                                                     wR2(reflections)=
R(reflections)= 0.0650( 2238)                        0.1730( 2902)

S = 1.223                    Npar= 107
```

The following ALERTS were generated. Each ALERT has the format
test-name_ALERT_alert-type_alert-level.
Click on the hyperlinks for more details of the test.

Alert level B

```
PLAT342_ALERT_3_B Low Bond Precision on  C-C Bonds ..............    0.02243 Ang.
```

Author Response: For organic-inorganic hybrid halide perovskites, thermal vibrations of organic molecules are strong at room temperature, especially in the presence of organic cations doping in this structure, which affect the abnormality of individual atoms and the precision of the C-C bond. We believed this problem will not affect the inorganic components we analyzed.

Alert level C

```
PLAT053_ALERT_1_C Minimum Crystal Dimension Missing (or Error) ...     Please Check
PLAT054_ALERT_1_C Medium  Crystal Dimension Missing (or Error) ...     Please Check
PLAT055_ALERT_1_C Maximum Crystal Dimension Missing (or Error) ...     Please Check
PLAT094_ALERT_2_C Ratio of Maximum / Minimum Residual Density ....       2.04 Report
PLAT230_ALERT_2_C Hirshfeld Test Diff for    F006     --C008     .        5.3 s.u.
PLAT242_ALERT_2_C Low    'MainMol' Ueq as Compared to Neighbors of       Sn00 Check
PLAT242_ALERT_2_C Low    'MainMol' Ueq as Compared to Neighbors of       C005 Check
PLAT242_ALERT_2_C Low    'MainMol' Ueq as Compared to Neighbors of       C008 Check
PLAT334_ALERT_2_C Small <C-C> Benzene Dist.   C005     -C009     .       1.36 Ang.
PLAT420_ALERT_2_C D-H Bond Without Acceptor  N004     --H00A     .     Please Check
PLAT906_ALERT_3_C Large K Value in the Analysis of Variance ......     13.174 Check
PLAT906_ALERT_3_C Large K Value in the Analysis of Variance ......      3.122 Check
PLAT911_ALERT_3_C Missing FCF Refl Between Thmin & STh/L=    0.600          8 Report
                3  1  2,   5  3  2,   8  2  3,   7  5  4,   9  3  4,   8  2  5,
                7  5  6,   9  3  6,
PLAT971_ALERT_2_C Check Calcd Resid. Dens.  1.38Ang From C009            2.37 eA-3
PLAT971_ALERT_2_C Check Calcd Resid. Dens.  0.97Ang From I002            1.86 eA-3
PLAT971_ALERT_2_C Check Calcd Resid. Dens.  1.06Ang From I002            1.71 eA-3
PLAT971_ALERT_2_C Check Calcd Resid. Dens.  1.01Ang From I003            1.66 eA-3
PLAT971_ALERT_2_C Check Calcd Resid. Dens.  1.02Ang From Sn00            1.59 eA-3
PLAT971_ALERT_2_C Check Calcd Resid. Dens.  1.05Ang From I003            1.56 eA-3
```

Alert level G

```
PLAT004_ALERT_5_G Polymeric Structure Found with Maximum Dimension          2 Info
PLAT005_ALERT_5_G No Embedded Refinement Details Found  in the CIF     Please Do !
PLAT007_ALERT_5_G Number of Unrefined Donor-H Atoms .............           3 Report
              H00A  H00B  H00C
PLAT083_ALERT_2_G SHELXL Second Parameter in WGHT  Unusually Large      20.25 Why ?
PLAT232_ALERT_2_G Hirshfeld Test Diff (M-X)  I003     --Sn00     .       12.4 s.u.
PLAT720_ALERT_4_G Number of Unusual/Non-Standard Labels .........          21 Note
              Sn00    I002    I003    N004    H00A    H00B    H00C    C005
              F006    C007    H00D    H00E    C008    C009    H009    C00A
              H00F    C00B    H00G    C00C    H00H
PLAT764_ALERT_4_G Overcomplete CIF Bond List Detected (Rep/Expd) .       1.25 Ratio
PLAT794_ALERT_5_G Tentative Bond Valency for Sn00      (II)      .       2.22 Info
```

```
PLAT910_ALERT_3_G Missing # of FCF Reflection(s) Below Theta(Min).          1 Note
                0  0  2,
PLAT912_ALERT_4_G Missing # of FCF Reflections Above STh/L=  0.600        264 Note
PLAT969_ALERT_5_G The 'Henn et al.' R-Factor-gap value ..........      3.759 Note
              Predicted wR2: Based on SigI**2  4.60 or SHELX Weight 14.14
PLAT978_ALERT_2_G Number C-C Bonds with Positive Residual Density.          1 Info
```

```
   0 ALERT level A = Most likely a serious problem - resolve or explain
   1 ALERT level B = A potentially serious problem, consider carefully
  19 ALERT level C = Check. Ensure it is not caused by an omission or oversight
  12 ALERT level G = General information/check it is not something unexpected

   3 ALERT type 1 CIF construction/syntax error, inconsistent or missing data
  16 ALERT type 2 Indicator that the structure model may be wrong or deficient
   5 ALERT type 3 Indicator that the structure quality may be low
   3 ALERT type 4 Improvement, methodology, query or suggestion
   5 ALERT type 5 Informative message, check
```

It is advisable to attempt to resolve as many as possible of the alerts in all categories. Often the minor alerts point to easily fixed oversights, errors and omissions in your CIF or refinement strategy, so attention to these fine details can be worthwhile. In order to resolve some of the more serious problems it may be necessary to carry out additional measurements or structure refinements. However, the purpose of your study may justify the reported deviations and the more serious of these should normally be commented upon in the discussion or experimental section of a paper or in the "special_details" fields of the CIF. checkCIF was carefully designed to identify outliers and unusual parameters, but every test has its limitations and alerts that are not important in a particular case may appear. Conversely, the absence of alerts does not guarantee there are no aspects of the results needing attention. It is up to the individual to critically assess their own results and, if necessary, seek expert advice.

Publication of your CIF in IUCr journals

A basic structural check has been run on your CIF. These basic checks will be run on all CIFs submitted for publication in IUCr journals (*Acta Crystallographica*, *Journal of Applied Crystallography*, *Journal of Synchrotron Radiation*); however, if you intend to submit to *Acta Crystallographica Section C* or *E* or *IUCrData*, you should make sure that full publication checks are run on the final version of your CIF prior to submission.

Publication of your CIF in other journals

Please refer to the *Notes for Authors* of the relevant journal for any special instructions relating to CIF submission.

PLATON version of 13/05/2024; check.def file version of 04/05/2024

Datablock exp_14795 - ellipsoid plot

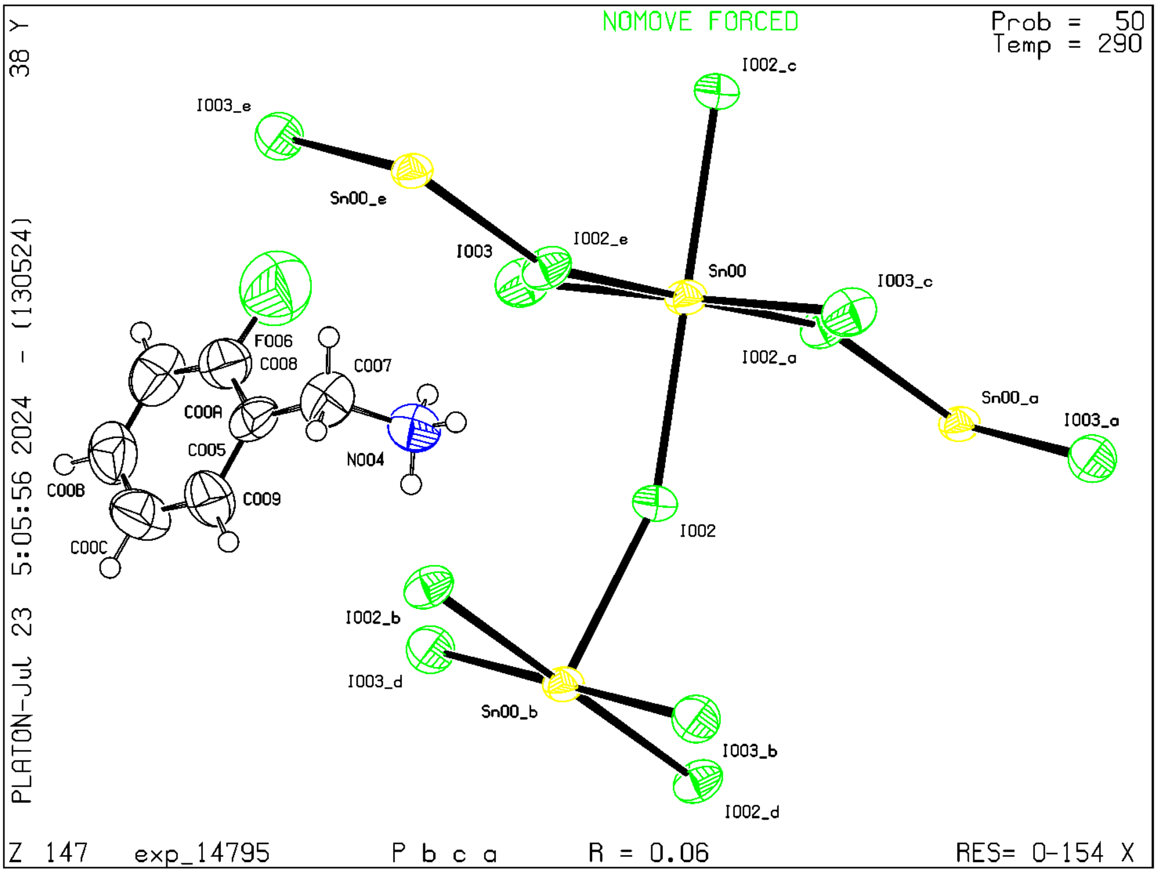

Supplement: Supplementary file 4 — Supplementary Data 1 [file (2FPMA)2SnI4 checkcif.pdf › crystal structure cif and checkcif/(2FPMA)2SnI4 checkcif.pdf]
