# Supplementary material for: Two-dimensional organic-inorganic hybrid perovskite quantum-well nanowires enabled by directional noncovalent intermolecular interactions
Source: Nat Commun. 2025 Mar 27;16:2997. doi: 10.1038/s41467-025-58166-x (PMC11950231; doi:10.1038/s41467-025-58166-x)

checkCIF/PLATON report

Structure factors have been supplied for datablock(s) HPipA2CdCl4

THIS REPORT IS FOR GUIDANCE ONLY. IF USED AS PART OF A REVIEW PROCEDURE FOR PUBLICATION, IT SHOULD NOT REPLACE THE EXPERTISE OF AN EXPERIENCED CRYSTALLOGRAPHIC REFEREE.

No syntax errors found. CIF dictionary Interpreting this report

Datablock: HPipA2CdCl4

Bond precision:	C-C = 0.0086 A		Wavelength=0.71073
Cell:	a=20.3740(14)	b=7.4322(5)	c=7.4956(4)
	alpha=90	beta=91.162(6)	gamma=90
Temperature:	290 K		

	Calculated	Reported
Volume	1134.78(12)	1134.78(12)
Space group	P 21/c	P 1 21/c 1
Hall group	-P 2ybc	-P 2ybc
Moiety formula	Cd Cl4, 2(C9 H12 N O2)	Cd Cl4, 2(C9 H12 N O2)
Sum formula	C18 H24 Cd Cl4 N2 O4	C18 H24 Cd Cl4 N2 O4
Mr	586.60	586.59
Dx,g cm-3	1.717	1.717
Z	2	2
Mu (mm-1)	1.460	1.460
F000	588.0	588.0
F000'	587.76	
h,k,lmax	28,10,10	27,10,10
Nref	3157	2839
Tmin,Tmax	0.719,0.919	0.575,1.000
Tmin'	0.632	

Correction method= # Reported T Limits: Tmin=0.575 Tmax=1.000
AbsCorr = GAUSSIAN

Data completeness= 0.899 Theta(max)= 29.456

R(reflections)= 0.0761(2338) wR2(reflections)= 0.2119(2839)

S = 1.072 Npar= 132

The following ALERTS were generated. Each ALERT has the format
test-name_ALERT_alert-type_alert-level.
Click on the hyperlinks for more details of the test.

Alert level B

```
PLAT971_ALERT_2_B Check Calcd Resid. Dens.  0.54Ang From Cd            3.30 eA-3
```

Author Response: The large positive calculated residual density on heavy atoms is usually caused by twining. However, checking by Platon does not find obvious twin laws. This might be due to the slightly worse crystal quality compared to other samples. For light atoms, this problem may arise from the organic cations doping in this structure and the highly disordered organic components.

Alert level C

```
PLAT094_ALERT_2_C Ratio of Maximum / Minimum Residual Density ....       2.14 Report
PLAT241_ALERT_2_C High   'MainMol' Ueq as Compared to Neighbors of       Cl02 Check
PLAT241_ALERT_2_C High   'MainMol' Ueq as Compared to Neighbors of       Cl03 Check
PLAT241_ALERT_2_C High   'MainMol' Ueq as Compared to Neighbors of         O1 Check
PLAT342_ALERT_3_C Low Bond Precision on  C-C Bonds ...............    0.00862 Ang.
PLAT420_ALERT_2_C D-H Bond Without Acceptor  N004     --H00A     .     Please Check
PLAT420_ALERT_2_C D-H Bond Without Acceptor  N004     --H00B     .     Please Check
PLAT420_ALERT_2_C D-H Bond Without Acceptor  N004     --H00C     .     Please Check
PLAT906_ALERT_3_C Large K Value in the Analysis of Variance ......      4.178 Check
PLAT911_ALERT_3_C Missing FCF Refl Between Thmin & STh/L=    0.600          9 Report
              -13  1  1, -12  5  1,  -9  5  1,  -6  4  1,  -4  1  1,  -4  2  1,
               -2  6  1,   5  2  1,   7  2  1,
PLAT971_ALERT_2_C Check Calcd Resid. Dens.  1.14Ang From Cl02          2.33 eA-3
```

Author Response: The large positive calculated residual density on heavy atoms is usually caused by twining. However, checking by Platon does not find obvious twin laws. This might be due to the slightly worse crystal quality compared to other samples. For light atoms, this problem may arise from the organic cations doping in this structure and the highly disordered organic components.

```
PLAT971_ALERT_2_C Check Calcd Resid. Dens.  1.11Ang From Cl03          2.01 eA-3
```

Author Response: The large positive calculated residual density on heavy atoms is usually caused by twining. However, checking by Platon does not find obvious twin laws. This might be due to the slightly worse crystal quality compared to other samples. For light atoms, this problem may arise from the organic cations doping in this structure and the highly disordered organic components.

```
PLAT971_ALERT_2_C Check Calcd Resid. Dens.  1.30Ang From Cd            1.76 eA-3
```

Author Response: The large positive calculated residual density on heavy atoms is usually caused by twining. However, checking by Platon does not find obvious twin laws. This might be due to the slightly worse crystal quality compared to other samples. For light atoms, this problem may arise from the organic cations doping in this structure and the highly disordered organic components.

```
PLAT972_ALERT_2_C Check Calcd Resid. Dens.  0.88Ang From Cl03          -1.52 eA-3
PLAT977_ALERT_2_C Check Negative Difference Density on H00F      .     -0.31 eA-3
PLAT977_ALERT_2_C Check Negative Difference Density on H006      .     -0.34 eA-3
PLAT977_ALERT_2_C Check Negative Difference Density on H00K      .     -0.50 eA-3
```

Alert level G

```
PLAT002_ALERT_2_G Number of Distance or Angle Restraints on AtSite          4 Note
PLAT003_ALERT_2_G Number of Uiso or U(i,j) Restrained non-H Atoms           8 Report
PLAT004_ALERT_5_G Polymeric Structure Found with Maximum Dimension          2 Info
PLAT007_ALERT_5_G Number of Unrefined Donor-H Atoms .............           3 Report
              H00A  H00B  H00C
PLAT012_ALERT_1_G N.O.K.   _shelx_res_checksum Found in CIF ......     Please Check
PLAT072_ALERT_2_G SHELXL First  Parameter in WGHT  Unusually Large       0.12 Report
PLAT083_ALERT_2_G SHELXL Second Parameter in WGHT  Unusually Large       6.74 Why ?
PLAT176_ALERT_4_G The CIF-Embedded .res File Contains SADI Records          2 Report
PLAT178_ALERT_4_G The CIF-Embedded .res File Contains SIMU Records          1 Report
PLAT186_ALERT_4_G The CIF-Embedded .res File Contains ISOR Records          3 Report
PLAT232_ALERT_2_G Hirshfeld Test Diff (M-X)  Cd01     --Cl03     .        5.1 s.u.
PLAT232_ALERT_2_G Hirshfeld Test Diff (M-X)  Cd       --Cl02     .        5.7 s.u.
PLAT232_ALERT_2_G Hirshfeld Test Diff (M-X)  Cd       --Cl02_c   .        6.0 s.u.
PLAT301_ALERT_3_G Main Residue  Disorder ..............(Resd    1)        32% Note
PLAT398_ALERT_2_G Deviating  C-O-C   Angle From 120 for O1       .      105.9 Degree
PLAT398_ALERT_2_G Deviating  C-O-C   Angle From 120 for O2       .      103.9 Degree
PLAT720_ALERT_4_G Number of Unusual/Non-Standard Labels ..........         25 Note
              Cd01    Cl02    Cl03    N004    H00A    H00B    H00C    C008
              H00D    H00E    C00A    H00F    H00G    C00E    H00H    C00C
              C00D    C006    H006    C007    C00B    H00I    C00F    H00J
              H00K
PLAT779_ALERT_4_G Suspect or Irrelevant (Bond) Angle(s) in CIF ...      14.10 Deg.
              CD01 -CL02 -CD      1_555   1_555   3_656 ......     #   16 Check
PLAT860_ALERT_3_G Number of Least-Squares Restraints .............         50 Note
PLAT910_ALERT_3_G Missing # of FCF Reflection(s) Below Theta(Min).          2 Note
                1  0  0,   2  0  0,
PLAT912_ALERT_4_G Missing # of FCF Reflections Above STh/L=  0.600        306 Note
PLAT933_ALERT_2_G Number of HKL-OMIT Records in Embedded .res File         10 Note
              -13  1  1, -12  5  1,  -9  5  1,  -6  4  1,  -4  1  1,  -4  2  1,
               -2  6  1,   2  0  0,   5  2  1,   7  2  1,
PLAT941_ALERT_3_G Average HKL Measurement Multiplicity ...........        2.7 Low
PLAT969_ALERT_5_G The 'Henn et al.' R-Factor-gap value ...........      3.298 Note
              Predicted wR2: Based on SigI**2  6.42 or SHELX Weight 19.76
PLAT978_ALERT_2_G Number C-C Bonds with Positive Residual Density.          0 Info
```

---

0 **ALERT level A** = Most likely a serious problem - resolve or explain

```
 1 ALERT level B = A potentially serious problem, consider carefully
17 ALERT level C = Check. Ensure it is not caused by an omission or oversight
25 ALERT level G = General information/check it is not something unexpected

 1 ALERT type 1 CIF construction/syntax error, inconsistent or missing data
26 ALERT type 2 Indicator that the structure model may be wrong or deficient
 7 ALERT type 3 Indicator that the structure quality may be low
 6 ALERT type 4 Improvement, methodology, query or suggestion
 3 ALERT type 5 Informative message, check
```

It is advisable to attempt to resolve as many as possible of the alerts in all categories. Often the minor alerts point to easily fixed oversights, errors and omissions in your CIF or refinement strategy, so attention to these fine details can be worthwhile. In order to resolve some of the more serious problems it may be necessary to carry out additional measurements or structure refinements. However, the purpose of your study may justify the reported deviations and the more serious of these should normally be commented upon in the discussion or experimental section of a paper or in the "special_details" fields of the CIF. checkCIF was carefully designed to identify outliers and unusual parameters, but every test has its limitations and alerts that are not important in a particular case may appear. Conversely, the absence of alerts does not guarantee there are no aspects of the results needing attention. It is up to the individual to critically assess their own results and, if necessary, seek expert advice.

Publication of your CIF in IUCr journals

A basic structural check has been run on your CIF. These basic checks will be run on all CIFs submitted for publication in IUCr journals (*Acta Crystallographica*, *Journal of Applied Crystallography*, *Journal of Synchrotron Radiation*); however, if you intend to submit to *Acta Crystallographica Section C* or *E* or *IUCrData*, you should make sure that full publication checks are run on the final version of your CIF prior to submission.

Publication of your CIF in other journals

Please refer to the *Notes for Authors* of the relevant journal for any special instructions relating to CIF submission.

PLATON version of 13/05/2024; check.def file version of 04/05/2024

Datablock HPipA2CdCl4 - ellipsoid plot

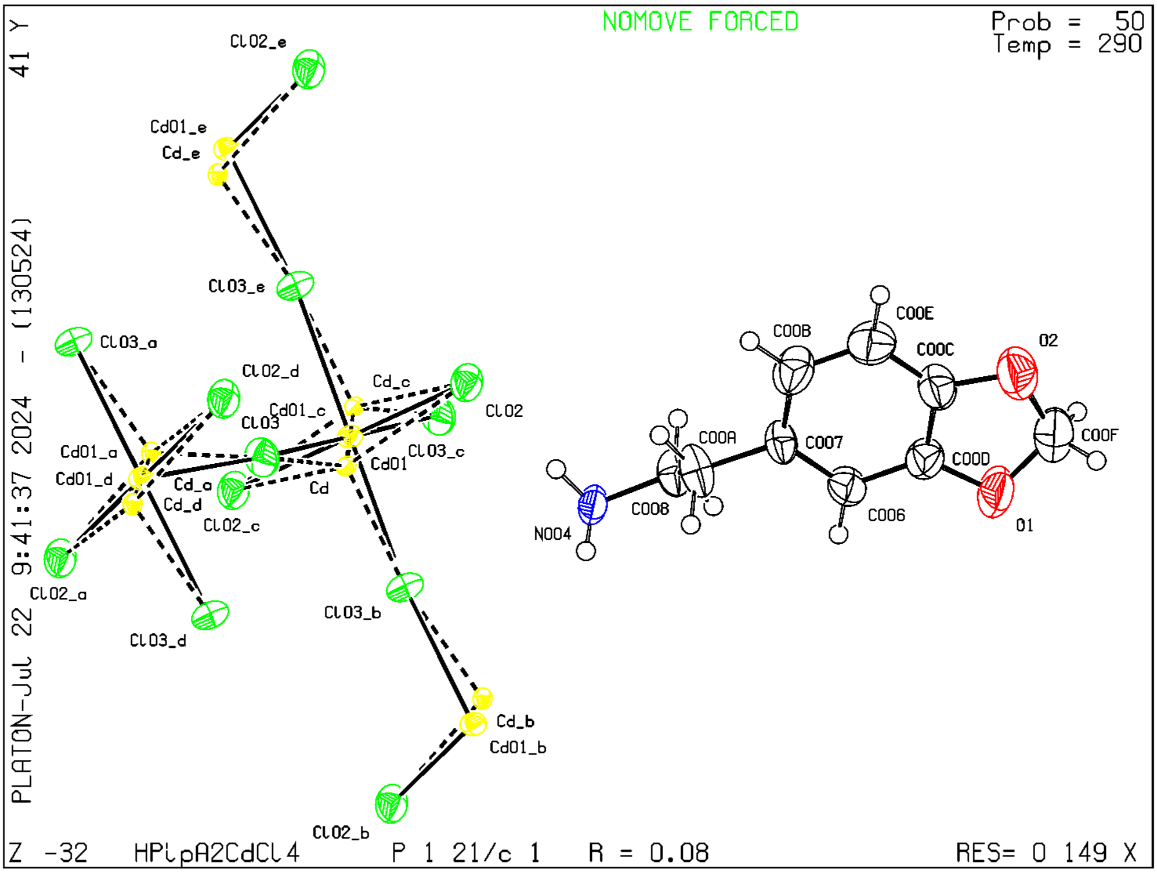

Supplement: Supplementary file 4 — Supplementary Data 1 [file (CH2O2PEA)2CdCl4.pdf › crystal structure cif and checkcif/(CH2O2PEA)2CdCl4.pdf]
