# Supplementary material for: Two-dimensional organic-inorganic hybrid perovskite quantum-well nanowires enabled by directional noncovalent intermolecular interactions
Source: Nat Commun. 2025 Mar 27;16:2997. doi: 10.1038/s41467-025-58166-x (PMC11950231; doi:10.1038/s41467-025-58166-x)

checkCIF/PLATON report

Structure factors have been supplied for datablock(s) exp_14790

THIS REPORT IS FOR GUIDANCE ONLY. IF USED AS PART OF A REVIEW PROCEDURE FOR PUBLICATION, IT SHOULD NOT REPLACE THE EXPERTISE OF AN EXPERIENCED CRYSTALLOGRAPHIC REFEREE.

No syntax errors found.   CIF dictionary   Interpreting this report

Datablock: exp_14790

Bond precision: C-C = 0.0375 A   Wavelength=0.71073

Cell:	a=14.723(4)	b=8.9747(18)	c=8.579(2)
	alpha=90	beta=99.65(3)	gamma=90
Temperature:	290 K		

	Calculated	Reported
Volume	1117.5(5)	1117.5(5)
Space group	P 21/c	P 1 21/c 1
Hall group	-P 2ybc	-P 2ybc
Moiety formula	I4 Sn, 2(C5 H14 N)	I4 Sn, 2(C5 H14 N)
Sum formula	C10 H28 I4 N2 Sn	C10 H28 I4 N2 Sn
Mr	802.65	802.63
Dx,g cm-3	2.385	2.385
Z	2	2
Mu (mm-1)	6.658	6.658
F000	728.0	728.0
F000'	723.16	
h,k,lmax	20,12,12	20,12,11
Nref	3252	2774
Tmin,Tmax		0.365,1.000
Tmin'		

Correction method= # Reported T Limits: Tmin=0.365 Tmax=1.000
AbsCorr = MULTI-SCAN

Data completeness= 0.853   Theta(max)= 29.984

R(reflections)= 0.0803(1744)   wR2(reflections)= 0.2714(2774)

S = 1.005   Npar= 81

The following ALERTS were generated. Each ALERT has the format
test-name_ALERT_alert-type_alert-level.
Click on the hyperlinks for more details of the test.

Alert level B

```
PLAT342_ALERT_3_B Low Bond Precision on  C-C Bonds ..............     0.0375 Ang.
```

Author Response: For organic-inorganic hybrid halide perovskites, thermal vibrations of organic molecules are strong at room temperature, especially in the presence of organic cations doping in this structure, which affect the abnormality of individual atoms and the precision of the C-C bond. We believed this problem will not affect the inorganic components we analyzed.

```
PLAT972_ALERT_2_B Check Calcd Resid. Dens.  0.89Ang From Sn00          -2.53 eA-3
```

Author Response: The large positive calculated residual density on heavy atoms is usually caused by twining. However, checking by Platon does not find obvious twin laws. This might be due to the slightly worse crystal quality compared to other samples. For light atoms, this problem may arise from the organic cations doping in this structure and the highly disordered organic components.

Alert level C

```
PLAT053_ALERT_1_C Minimum Crystal Dimension Missing (or Error) ...     Please Check
PLAT054_ALERT_1_C Medium  Crystal Dimension Missing (or Error) ...     Please Check
PLAT055_ALERT_1_C Maximum Crystal Dimension Missing (or Error) ...     Please Check
PLAT084_ALERT_3_C High wR2 Value (i.e. > 0.25) ..................       0.27 Report
PLAT242_ALERT_2_C Low    'MainMol' Ueq as Compared to Neighbors of      Sn00 Check
PLAT260_ALERT_2_C Large Average Ueq of Residue Including      N004      0.141 Check
PLAT360_ALERT_2_C Short  C(sp3)-C(sp3) Bond  C6       - C8       .       1.37 Ang.
PLAT420_ALERT_2_C D-H Bond Without Acceptor  N004     --H00A     .     Please Check
PLAT420_ALERT_2_C D-H Bond Without Acceptor  N004     --H00B     .     Please Check
PLAT420_ALERT_2_C D-H Bond Without Acceptor  N004     --H00C     .     Please Check
PLAT906_ALERT_3_C Large K Value in the Analysis of Variance ......      3.351 Check
PLAT911_ALERT_3_C Missing FCF Refl Between Thmin & STh/L=    0.600         31 Report
               -2  0  2,  -2  1  2,  -3  2  3,  -1  6  5,   1  6  5,   3  5  5,
               -4  4  6,  -4  6  6,  -3  5  6,  -2  5  6,   0  7  6,   2  6  6,
                2  7  6,   3  5  6,   3  6  6,  -5  3  7,  -5  4  7,  -4  5  7,
               -4  6  7,  -3  6  7,   3  7  7,   6  6  7,  -6  1  9,  -6  0 10,
               -6  1 10,  -5  2 10,  -4  0 10,  -4  1 10,  -4  2 10,  -2  0 10,
               -1  0 10,
PLAT918_ALERT_3_C Reflection(s) with I(obs) much Smaller I(calc) .          1 Check
PLAT939_ALERT_3_C Large Value of Not (SHELXL) Weight Optimized S .      19.26 Check
PLAT971_ALERT_2_C Check Calcd Resid. Dens.  2.47Ang From N004           2.30 eA-3
PLAT971_ALERT_2_C Check Calcd Resid. Dens.  1.13Ang From C5             2.11 eA-3
PLAT971_ALERT_2_C Check Calcd Resid. Dens.  1.09Ang From N004           1.99 eA-3
PLAT971_ALERT_2_C Check Calcd Resid. Dens.  1.97Ang From I002           1.91 eA-3
```

```
PLAT971_ALERT_2_C Check Calcd Resid. Dens.  1.33Ang From I002           1.60 eA-3
PLAT971_ALERT_2_C Check Calcd Resid. Dens.  0.95Ang From I002           1.54 eA-3
PLAT971_ALERT_2_C Check Calcd Resid. Dens.  1.60Ang From I003           1.52 eA-3
PLAT972_ALERT_2_C Check Calcd Resid. Dens.  0.90Ang From Sn00          -2.04 eA-3
```

Author Response: The large positive calculated residual density on heavy atoms is usually caused by twining. However, checking by Platon does not find obvious twin laws. This might be due to the slightly worse crystal quality compared to other samples. For light atoms, this problem may arise from the organic cations doping in this structure and the highly disordered organic components.

```
PLAT972_ALERT_2_C Check Calcd Resid. Dens.  1.42Ang From Sn00          -1.61 eA-3
```

Author Response: The large positive calculated residual density on heavy atoms is usually caused by twining. However, checking by Platon does not find obvious twin laws. This might be due to the slightly worse crystal quality compared to other samples. For light atoms, this problem may arise from the organic cations doping in this structure and the highly disordered organic components.

```
PLAT972_ALERT_2_C Check Calcd Resid. Dens.  1.53Ang From I003          -1.60 eA-3
```

Author Response: The large positive calculated residual density on heavy atoms is usually caused by twining. However, checking by Platon does not find obvious twin laws. This might be due to the slightly worse crystal quality compared to other samples. For light atoms, this problem may arise from the organic cations doping in this structure and the highly disordered organic components.

```
PLAT972_ALERT_2_C Check Calcd Resid. Dens.  1.75Ang From I003          -1.59 eA-3
```

Author Response: The large positive calculated residual density on heavy atoms is usually caused by twining. However, checking by Platon does not find obvious twin laws. This might be due to the slightly worse crystal quality compared to other samples. For light atoms, this problem may arise from the organic cations doping in this structure and the highly disordered organic components.

```
PLAT972_ALERT_2_C Check Calcd Resid. Dens.  0.71Ang From I002          -1.58 eA-3
```

Author Response: The large positive calculated residual density on heavy atoms is usually caused by twining. However, checking by Platon does not find obvious twin laws. This might be due to the slightly worse crystal quality compared to other samples. For light atoms, this problem may arise from the organic cations doping in this structure and the highly disordered organic components.

```
PLAT972_ALERT_2_C Check Calcd Resid. Dens.  1.15Ang From I002          -1.55 eA-3
```

Author Response: The large positive calculated residual density on heavy atoms is usually caused by twining. However, checking by Platon does not find obvious twin laws. This might be due to the slightly worse crystal quality compared to other samples. For light atoms, this problem may arise from the organic cations doping in this structure and the highly disordered organic components.

```
PLAT977_ALERT_2_C Check Negative Difference Density on H6B       .      -0.67 eA-3
PLAT977_ALERT_2_C Check Negative Difference Density on H8B       .      -0.81 eA-3
```

Alert level G

```
PLAT002_ALERT_2_G Number of Distance or Angle Restraints on AtSite         6 Note
PLAT003_ALERT_2_G Number of Uiso or U(i,j) Restrained non-H Atoms          7 Report
PLAT004_ALERT_5_G Polymeric Structure Found with Maximum Dimension         2 Info
PLAT005_ALERT_5_G No Embedded Refinement Details Found  in the CIF     Please Do !
PLAT007_ALERT_5_G Number of Unrefined Donor-H Atoms .............          3 Report
              H00A  H00B  H00C
PLAT072_ALERT_2_G SHELXL First  Parameter in WGHT  Unusually Large       0.18 Report
PLAT232_ALERT_2_G Hirshfeld Test Diff (M-X)  I003     --Sn00     .        8.3 s.u.
PLAT720_ALERT_4_G Number of Unusual/Non-Standard Labels ..........         11 Note
              Sn00    I002    I003    N004    H00A    H00B    H00C    C005
              H00D    H00E    H00F
PLAT764_ALERT_4_G Overcomplete CIF Bond List Detected (Rep/Expd) .       1.38 Ratio
PLAT794_ALERT_5_G Tentative Bond Valency for Sn00      (II)      .       2.49 Info
PLAT860_ALERT_3_G Number of Least-Squares Restraints .............         61 Note
PLAT910_ALERT_3_G Missing # of FCF Reflection(s) Below Theta(Min).          1 Note
                1  0  0,
PLAT912_ALERT_4_G Missing # of FCF Reflections Above STh/L=  0.600        440 Note
PLAT969_ALERT_5_G The 'Henn et al.' R-Factor-gap value ...........      3.325 Note
              Predicted wR2: Based on SigI**2  8.16 or SHELX Weight 27.01
PLAT978_ALERT_2_G Number C-C Bonds with Positive Residual Density.          0 Info
```

---

0 **ALERT level A** = Most likely a serious problem - resolve or explain
2 **ALERT level B** = A potentially serious problem, consider carefully
29 **ALERT level C** = Check. Ensure it is not caused by an omission or oversight
15 **ALERT level G** = General information/check it is not something unexpected

3 ALERT type 1 CIF construction/syntax error, inconsistent or missing data

```
27 ALERT type 2 Indicator that the structure model may be wrong or deficient
 8 ALERT type 3 Indicator that the structure quality may be low
 3 ALERT type 4 Improvement, methodology, query or suggestion
 5 ALERT type 5 Informative message, check
```

It is advisable to attempt to resolve as many as possible of the alerts in all categories. Often the minor alerts point to easily fixed oversights, errors and omissions in your CIF or refinement strategy, so attention to these fine details can be worthwhile. In order to resolve some of the more serious problems it may be necessary to carry out additional measurements or structure refinements. However, the purpose of your study may justify the reported deviations and the more serious of these should normally be commented upon in the discussion or experimental section of a paper or in the "special_details" fields of the CIF. checkCIF was carefully designed to identify outliers and unusual parameters, but every test has its limitations and alerts that are not important in a particular case may appear. Conversely, the absence of alerts does not guarantee there are no aspects of the results needing attention. It is up to the individual to critically assess their own results and, if necessary, seek expert advice.

Publication of your CIF in IUCr journals

A basic structural check has been run on your CIF. These basic checks will be run on all CIFs submitted for publication in IUCr journals (*Acta Crystallographica*, *Journal of Applied Crystallography*, *Journal of Synchrotron Radiation*); however, if you intend to submit to *Acta Crystallographica Section C* or *E* or *IUCrData*, you should make sure that full publication checks are run on the final version of your CIF prior to submission.

Publication of your CIF in other journals

Please refer to the *Notes for Authors* of the relevant journal for any special instructions relating to CIF submission.

PLATON version of 13/05/2024; check.def file version of 04/05/2024

Datablock exp_14790 - ellipsoid plot

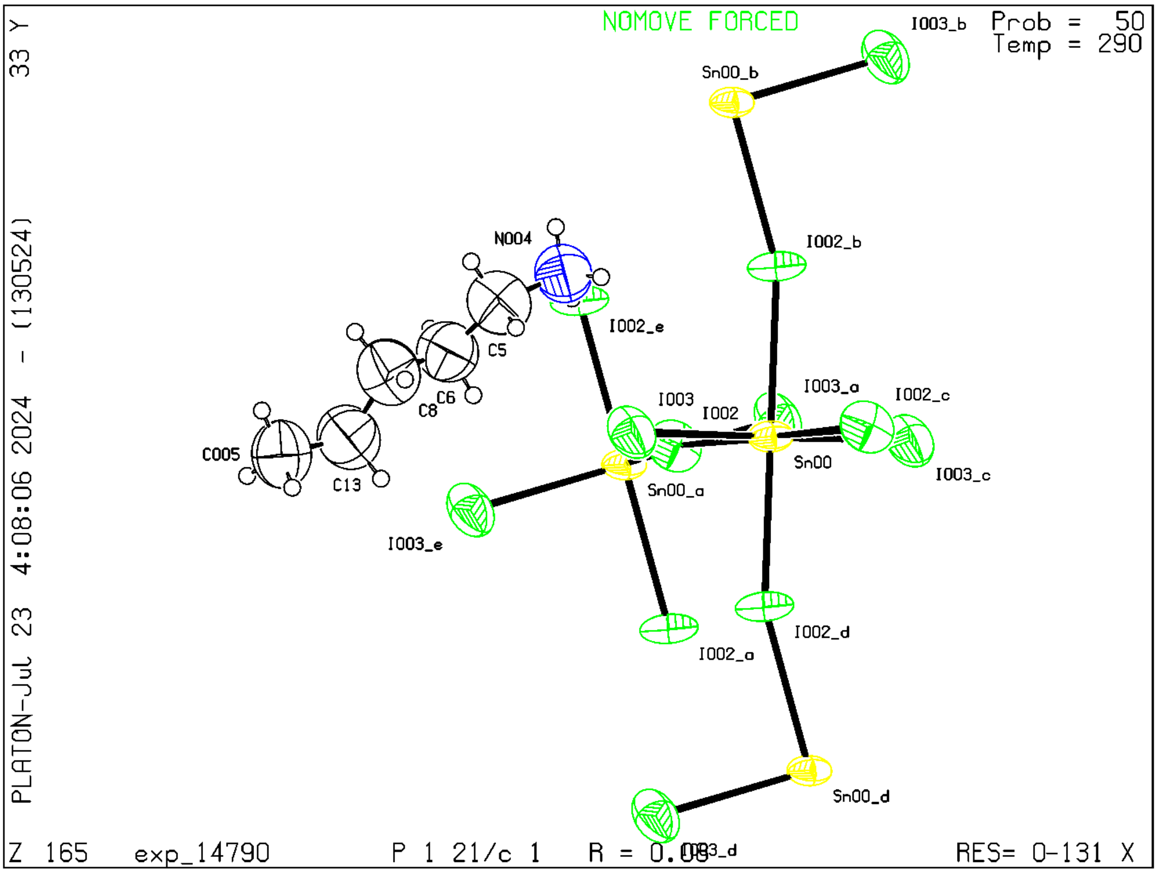

Supplement: Supplementary file 4 — Supplementary Data 1 [file (PA)2SnI4 checkcif.pdf › crystal structure cif and checkcif/(PA)2SnI4 checkcif.pdf]
